# Dynamic Prediction of Day Mortality in Patients With Trauma Using a Hybrid Neural Network Model: Model Development and Evaluation Study

**DOI:** 10.2196/86202

**Published:** 2026-09-16

**Authors:** Andreas Skov Millarch, Haytham Kaafarani, Ibrahim Chamseddine, Fredrik Folke, Søren Steeman Rudolph, Martin Sillesen

**Affiliations:** 1 Rigshospitalet Copenhagen Denmark; 2 Division of Trauma, Emergency Surgery and surgical critical care Mass General Brigham Boston, MA United States; 3 Harvard University boston, MA United States; 4 Department of Radiation Oncology Mass General Brigham Boston, MA United States; 5 Gentofte Hospital Gentofte Denmark; 6 University of Copenhagen Copenhagen, Capital Region Denmark

**Keywords:** dynamic, electronic health records, hybrid neural network, machine learning risk prediction, patient trajectory modeling, temporal validation, transformer, trauma risk assessment

## Abstract

**Background:**

The trajectory of a patient with trauma is often complex and nonlinear. Real-time estimation of the mortality risk from prehospital care to discharge is critical for point-of-care decision-making and for benchmarking the quality of care. Conventional risk assessment systems in trauma are simple and data-sparse, leaving potential for harvesting available data for personalized risk assessments accounting for developing patient states.

**Objective:**

This study aims to create an AI risk prediction model for 30-day mortality in patients with trauma capable of performing predictions at any time point through treatment phases from prehospital to discharge.

**Methods:**

Data on pre- and in-hospital care of patients with trauma treated in Denmark, Capital Region between 2017 and 2024 were used. Demographic and comorbidity-specific variables were structured as tabular data. Temporal data including vitals, laboratory test results, and medications were structured as sequences with temporally determined dynamic bin sizes. Trajectories were censored before the outcome event. Across continuous input types, scaling and normalization parameters were fitted on observed values only, and missing values were handled through zero-imputation with auxiliary indicator channels enabling the model to distinguish observed from absent measurements. Missing values across categorical input types were mapped to a reserved token with a learned embedding. The model architecture combines both tabular and sequential input data. A unified input is processed by multiple layers of transformer encoders before being passed into a neural network for binary classification. Model performance was assessed using area under the receiver operating characteristic curve (AUROC) and area under the precision-recall curve (AUPRC) on a holdout dataset comprising 20% of the total data.

**Results:**

A total of 9496 patients were included. The model achieved an AUROC of 0.962 (95% CI 0.930-0.994) and an AUPRC of 0.655 (95% CI 0.545-0.765) predicting 30-day mortality in the holdout evaluation set using full-length trajectories, comprising 1829 patients. In active-cohort evaluation, the model achieved an AUROC of 0.905 (95% CI 0.856-0.953) at 1 hour from first patient contact, demonstrating early discriminative capability from the prehospital phase onward. The model outperformed both Revised Trauma Score (mean ΔAUROC +0.297; 50/54 time points significant) and Trauma and Injury Severity Score (mean ΔAUROC +0.169; 46/54 time points significant).

**Conclusions:**

We designed a dynamic, automated model that allows risk prediction at any point in time during nonlinear trajectory of the patient with trauma. This study demonstrates that a hybrid neural network model, trained on automatically extracted electronic health record data, can predict 30-day all-cause mortality in patients with trauma with strong discrimination across the care trajectory. The model could be used as decision support for triage, patient deterioration alerts, bedside decision-making, and family counseling. These findings support the feasibility of sequential modeling for trauma risk prediction.

## Introduction

The trajectory of a patient with trauma is often complex and nonlinear, creating a highly dynamic data flow that has the potential to predict clinically relevant outcomes [1,2]. This data flow starts from the prehospital setting, continues through to hospital treatment and discharge, encompassing a wide range of parameters, including physiological measurements, clinical assessments, treatment interventions, and patient responses. Real-time estimation of the mortality risk from prehospital care to discharge is critical for decision-making and benchmarking the quality of care. First, it informs crucial decision-making processes, allowing health care providers to adapt treatment strategies promptly and allocate resources efficiently. Second, it serves as a valuable tool for benchmarking the quality of care across different trauma centers and systems. As health care systems increasingly leverage big data and machine learning (ML) technologies, the integration of dynamic risk assessment models into clinical practice holds promise for significantly improving patient outcomes in trauma care [3].

Conventionally, mortality risk estimation in trauma relies only on summarized information about the patient state at a certain time point, for example, the first value of systolic blood pressure, or by aggregation, such as the lowest systolic blood pressure. A prime example is the widely used Trauma and Injury Severity Score (TRISS) [4], which is calculated by trauma type (blunt or penetrating), age group, Revised Trauma Score (RTS) [5], and Injury Severity Score (ISS) [6]. The RTS, a component of TRISS, is calculated using the initial values of Glasgow Coma Score (GCS) [7], systolic blood pressure, and respiratory rate. While these established scoring systems have demonstrated clinical use, they have significant limitations. By reducing complex time-series data to single values, they inherently discard a wealth of potentially valuable information about the patient’s evolving condition. This approach fails to capture the dynamic nature of trauma progression and response to treatment, potentially overlooking critical trends and patterns that could inform more accurate risk assessments and guide clinical decision-making. As health care technology advances, there is a growing opportunity to leverage the continuous stream of patient data being collected, potentially enhancing the precision and timeliness of mortality risk predictions in trauma care.

ML models offer significant advantages in predicting trauma outcomes, particularly in their ability to handle complex data relationships and numerous input features. These models can capture nonlinear relationships between variables that are often challenging to detect using classical statistical approaches. This capability is especially valuable in trauma care, where patient outcomes may depend on intricate interactions between multiple factors. Previous studies have demonstrated superior predictive performance using ML compared to TRISS in predicting mortality [8,9] and the potential to predict posttrauma complications [10]. In contrast to most previous studies, which use static information, the model architecture used in this study combines tabular and time-series data through a sequential data fusion approach. This adds a sense of timing and context to the model, which currently can be harvested using attention, enhancing its ability to capture dynamic patterns and relationships [11]. Using a modeling approach that can capture intricate interactions between multiple factors with a sense of timing and context of changes in treatment and patient states may improve predictive performance.

The overall concept is depicted in Figure 1. Using data automatically extracted from prehospital and in-hospital electronic health record (EHR) for ease of use in an acute clinical setting, we aimed to create a dynamic AI risk prediction model for 30-day mortality in patients with trauma, capable of performing dynamic predictions at any time point, recalculating predictions at each timestep, through treatment phases from prehospital to discharge fully using the array of data generated as patients advance through treatment trajectories. We use the term “dynamic” to refer to this sequential updating of predictions with accumulating input data. In the framing taxonomy of Lauritsen et al [12], the model is left-aligned with a fixed prediction window and a variable-length observation window that expands as clinical data accumulates.

**Figure 1:**
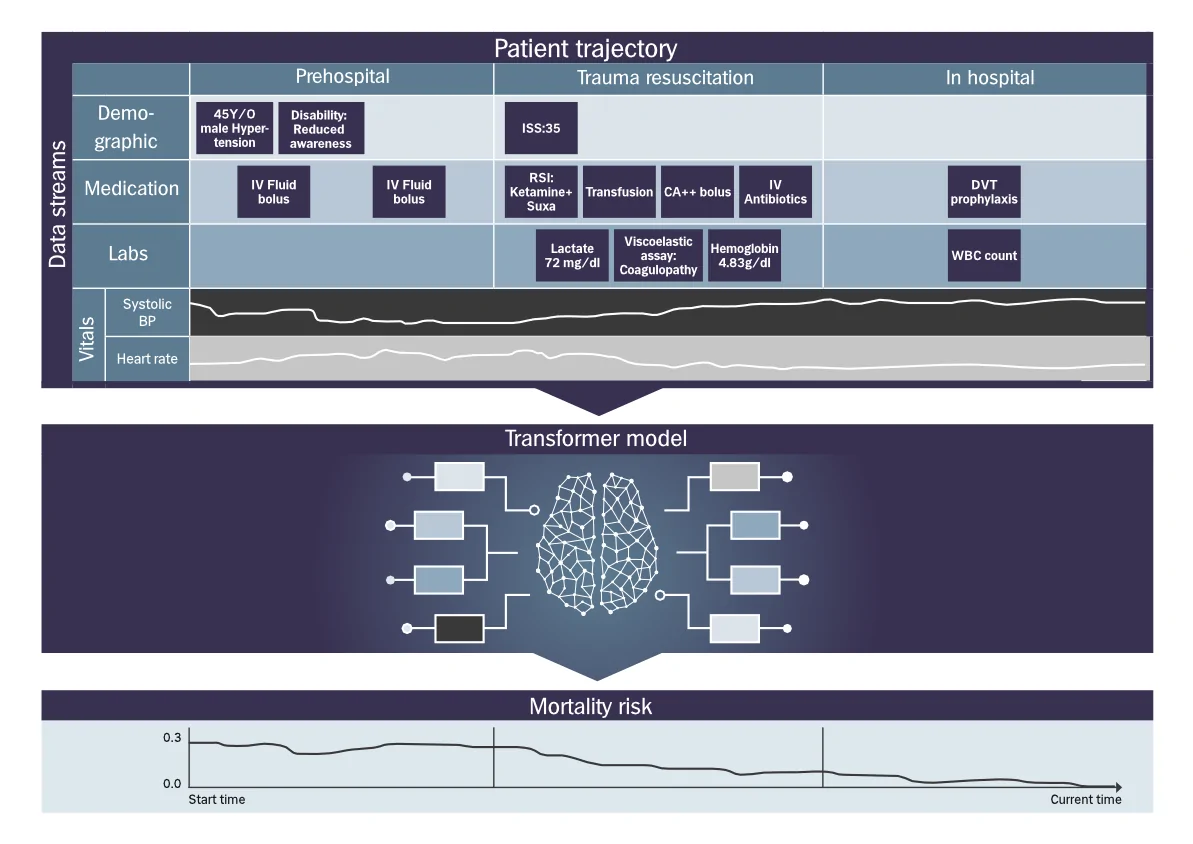
An example of a patient trajectory consisting of several phases and different data streams feeding into our model as they are created. Thus, displaying the clinical evolution of a patient and the dynamic development in mortality risk estimation over time from prehospital till discharge. BP: blood pressure; DVT: deep venous thrombosis; IV: intravenous; RSI: rapid sequence induction; WBC: white blood cell count.

Potential clinical applications of the proposed model include triaging, urgency awareness in the trauma bay, decision support for treatment strategy in the operative setting, structured communication during transitions between treatment phases, patient family counseling, and clinical quality work.

## Methods

### Ethics Statement

The study was approved by the Danish Patient Safety Board (Styrelsen for Patientsikkerhed, approval #31-1521), and the Danish Capital Regions Data Safety Board (Videncenter for Dataanmeldelser, approval #P-2020-180). In compliance with Danish legislation on health care research, patient consent was not required due to the retrospective and deidentified nature of the dataset and thus not obtained.

### Data Sources and Inclusion

The study used retrospective data from pre- and in-hospital EHRs from patients with trauma treated at any of the 6 acute care hospitals in the Capital Region of Denmark from 2017 to June 2024. This region covers 1.9 million inhabitants, where health care (including acute care services) is offered free of charge for all citizens. Prehospital triage criteria to either a local acute care hospital or the region’s single level I trauma center (Rigshospitalet) are based on clinical guidelines (Figure S1 in Multimedia Appendix 1).

Prehospital EHR data were obtained through the Emergency Medical Services, Capital Region of Denmark. In-hospital EHR data were provided through the Capital Region Research Data Analysis Platform.

Patients were identified by treatment through an in-hospital EHR trauma call registration (Danish procedure code BWST1F). We included patients of all ages who were present in both the pre- and in-hospital EHR with any type of trauma and mechanism of injury. Patients declared dead on scene were excluded from the dataset. Of note, trauma team activation criteria follow local hospital guidelines and are therefore not standardized.

### Outcome

The model predicts 30-day all-cause mortality calculated from the trauma date. The date of death was obtained from the EHR, which is updated with information on both in- and out-of-hospital deaths using the Danish Central Person Registry. As such, the EHR data also provide information on postdischarge mortality.

To prevent data leakage from perimortem clinical patterns (eg, terminal vital sign deterioration or treatment withdrawal), the trajectory end time of deceased patients was truncated prior to time binning and feature extraction. A proportional masking strategy was applied, stratified by trajectory duration: the final 10% was removed for short trajectories (≤6 hours), 5% for medium trajectories (6-72 hours), and 2% for long trajectories (>72 hours). This ensures more aggressive masking for shorter stays, where perimortem patterns constitute a larger fraction of the observed data. A minimum postmasking trajectory duration of 30 minutes was enforced. Since masking is applied before all downstream processing, subsequent feature aggregation and normalization only reflect the truncated observation window. Surviving patients are unaffected.

### Dataset Construction

Trauma cases were identified as defined above (all patients meeting trauma team activation criteria at any acute care hospital in the Capital Region of Denmark). Demographics and comorbidity variables were structured as tabular data and obtained from the EHR. Vital signs, laboratory test results, and medication were structured as sequential time-series data.

Prehospital data encompassed continuous measurements of vital signs, that is, blood pressure, heart rate, and oxygen saturation, and GCS and airway, breathing, circulation, and disability (ABCD) assessments. In-hospital EHR data consisted of vital signs; GCS; medication groupings (ie, opioids, antibiotics, neurological drugs, antithrombotic drugs, cardiovascular drugs, infusions, anesthetics, diuretics, insulin, hemostatics, and blood transfusions); laboratory results (ie, lactate, base excess, hemoglobin, leukocyte count, and thromboelastogram [Haemonetics Mount Juliet] measures); and admission, discharge, and transfer (ADT) events grouped as operation room, intensive care unit (ICU), and inpatient ward. The underlying Anatomical Therapeutic Chemical (ATC) codes for each medication group are presented in Table S1 in Multimedia Appendix 1. Laboratory test groupings and underlying variables are described in Table S2 in Multimedia Appendix 1.

The trajectory beginning was marked using the first available time stamp from the prehospital EHR for each trauma case. The in-hospital starting and ending time points were marked for each case using in-hospital data on ADT events. Using temporally determined binning frequencies for the data segments with increasing bin size over time up to 30 days allowed using a higher granularity of data at the beginning of patient trajectories, where the patient state is also more dynamic, while allowing longer sequences within model architectural limits. Binning frequencies for each segment are presented in Table S3 in Multimedia Appendix 1.

The segments were used to map vital signs, laboratory test results, ISS, and medications across the entire patient trajectory when available. Vital signs, GCS, and laboratory tests were represented at their absolute values, and medication groups were multihot-encoded in each segment. Vital signs were summarized in each segment as the mean and SD of the binned values. Laboratory tests were summarized using the maximum value in each segment.

Diagnosis codes, based on the *ICD* (*International Classification of Diseases*), prior to the trauma event were used to calculate the Elixhauser Comorbidity Index (ECI) [13] using the comorbidity R package [14]. Patients without prior diagnosis records were assigned an ECI of zero, as absence from the diagnosis registry was interpreted as no documented comorbidities rather than a missing value. Diagnosis codes registered during the hospital stay were used for estimating ISS using ICDPIC-R [15], as previously demonstrated to be feasible in a Danish population with trauma [16]. Moreover, ISS registrations were extracted from free-text clinical notes using regular expression pattern matching. To prevent data leakage, ISS was entered into the model only when available, based on the latest relevant diagnosis registration date or note last-edited time stamp, respectively. This ensures the model has access only to information that would be available at each prediction time point in a prospective setting, at the cost of potential input lag relative to the true time of clinical assessment.

Initial prehospital assessments of ABCD [17] were also treated as tabular data using the predetermined values available to emergency service providers nationally on their EHR interface. The classifications are presented in Table S4 in Multimedia Appendix 1. In the case of level of affectedness, these categories are not objectively defined but are based on the judgment of the prehospital provider using a mix of established reference ranges, patient-specific factors, and clinical context to decide whether a particular category is abnormal. Therefore, this model input feature should be regarded as a proxy for the clinician’s level of concern regarding each of the ABCD categories.

### Preprocessing

The complete trauma dataset was split into a training dataset and a holdout dataset. The holdout dataset was split temporally, comprising the newest 20 percent of the complete data, and was only used after model training was completed for evaluation purposes to ensure proper model generalization. All normalization statistics, both tabular and time series, were computed on the training set and applied to the holdout set without refitting, preventing data leakage.

For the tabular dataset, continuous features were normalized using an adaptive scaling approach where the transformation method (standard, robust, quantile, or power transform) was selected per variable based on skewness and kurtosis. Age and weight used standard scaling while ECI and height used quantile scaling. For continuous static variables with missing values (height and weight), scaling parameters were fitted on observed values only, excluding missing entries. After scaling, remaining missing values were filled with zero in the standardized space, and a binary indicator variable was created per variable, enabling the model to distinguish observed measurements from absent ones. Categorical features were integer-encoded with a per-variable vocabulary, where missing or unknown values were mapped to a reserved index-zero token with a learned embedding, enabling the model to learn a dedicated representation for missingness without requiring a separate indicator variable.

Time-series data exhibit 2 forms of absence: *missingness* (unrecorded measurements within a patient’s trajectory) and *padding* (positions beyond the trajectory end point). We distinguish these explicitly: trajectory lengths are derived from the data, and a binary padding mask identifies positions beyond each patient’s trajectory boundary.

Per-channel normalization (adaptive *z*-score, robust, or quantile transform selected per channel based on skewness and kurtosis) is fitted exclusively on observed values, excluding both missing and padding positions. Following normalization, measured values are approximately standard normal, while both missing and padding positions are imputed as zero. This zero-imputation scheme leverages the postnormalization distribution: since measured values are centered at zero with unit variance, the zero token represents the channel-specific population means, providing an uninformative but numerically stable default. An auxiliary channel for binary data presence indication is appended to the input tensor and excluded from normalization, enabling the model to distinguish observed measurements from imputed zeros. For categorical time series, a multi–hot count encoding is applied per time bin, where absence of a category is naturally represented as zero counts, requiring no separate imputation.

Per-channel normalization is fitted exclusively on observed values, excluding both missing and padding positions. Categorical time series (medications, procedures, and clinical events) use multi–hot count encoding per time bin, where absence is naturally represented as zero counts.

### Training Process

The complete dataset was temporally split into a training dataset (80%) and a holdout dataset (most recent 20%), with the holdout used only for final evaluation. The training dataset, hereafter referred to as the development dataset, was further split into 80% training and 20% internal validation folds during cross-validation [18] using 5 splits (illustrated in Figure S2 in Multimedia Appendix 1). For 50 trials, the folds were randomized but stratified on outcome prevalence. Random rather than temporal fold assignment was chosen so that each fold’s training partition spans the full temporal range of the development dataset, matching the conditions under which the final model is trained on the full development dataset. During this process (Figure 2), a range of hyperparameters was optimized against maximizing the sum of area under the precision-recall curve (AUPRC) across all folds. Using the optimized hyperparameters, the final model was trained on the full development dataset before evaluation on the temporally distinct holdout set.

**Figure 2:**
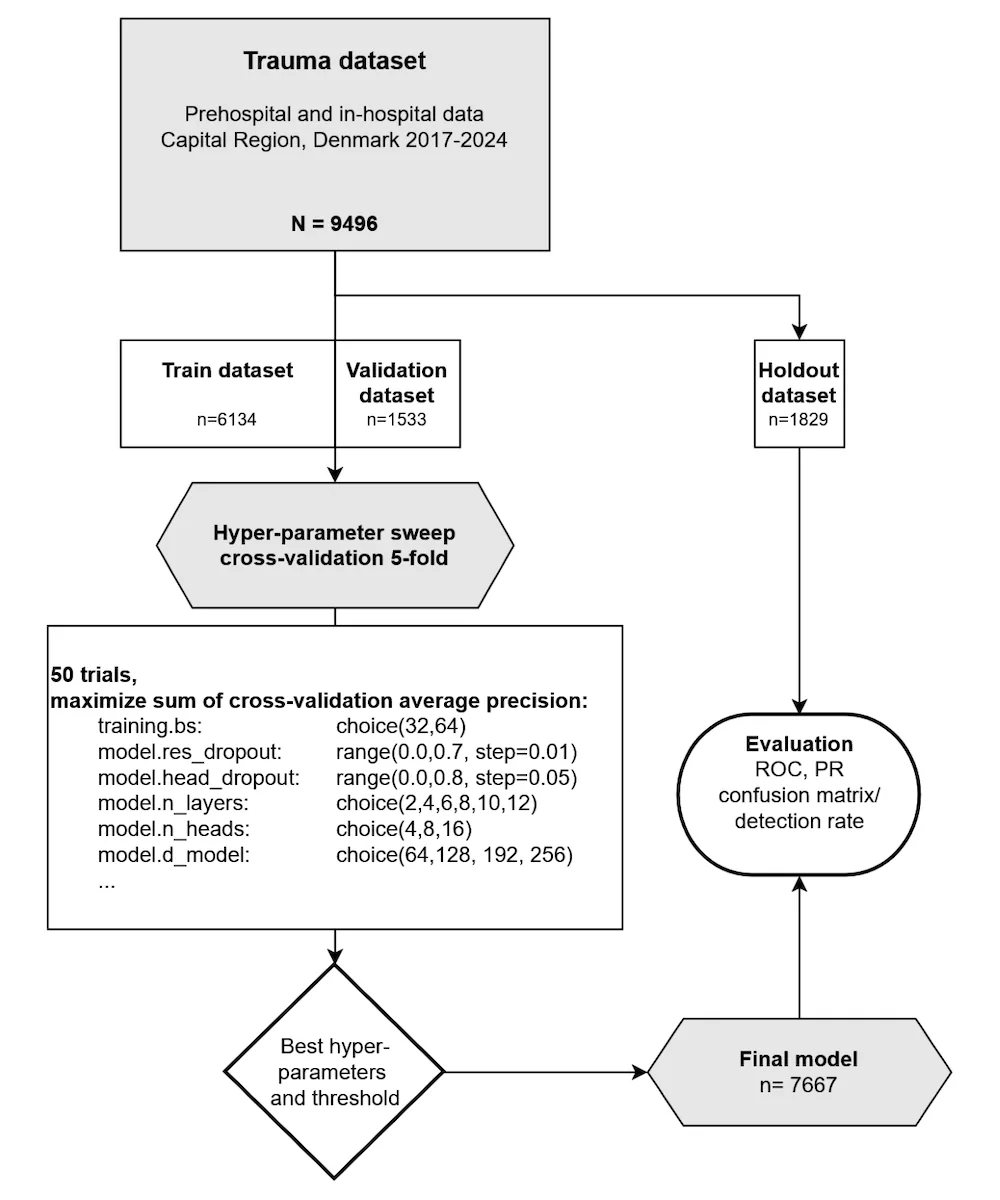
The overall training process displaying how optimal hyperparameters and thresholds were determined through hyperparameter sweeping over 50 trials each of 5-fold cross-validation, optimizing for the sum of area under the precision-recall curve for the internal folds and finally evaluating performance on the holdout dataset using receiver operating characteristic (ROC), precision-recall curve (PR), and confusion matrices. Each trial optimized parameters including: batch size (bs), residual dropout fraction (res_dropout), the model head dropout fraction (head_dropout), number of transformer encoder layers (n_layers), number of attention heads (n_heads), and model dimension (d_model).

### Model Architecture

Our model used a combination of static contextual features and sequential input data. We expanded on the timeseries-tabular-transformer [19], an adaptation of the TabTransformer [20] architecture designed for time-series and tabular data fusion. This hybrid neural network approach capitalizes on the attention mechanism’s strengths for processing sequential data while incorporating tabular patient information to contextualize the representation.

The transformer encoder applies causal masking, ensuring each temporal position attends only to itself and preceding timesteps, preventing information leakage from future observations. A shared-weight per-timestep prediction head produces independent mortality risk estimates at each temporal position, enabling continuous risk monitoring throughout the patient trajectory. Attention masking is used to ignore end-of-sequence padding.

The model architecture is elaborated in Appendix S1 in Multimedia Appendix 1. Implementations are listed in Appendix S2 in Multimedia Appendix 1.

### Performance Evaluation Metrics

To evaluate performance, we used a receiver operating characteristic (ROC) curve and a precision-recall (PR) curve. The ROC curve was summarized with the area under the receiver operating characteristic curve (AUROC), and for PR we calculated the AUPRC. CIs were calculated by the DeLong method for AUROC and bootstrapping for AUPRC.

Following the framing taxonomy for ML risk prediction proposed by Lauritsen et al [12], the model uses a left-aligned structure with a fixed prediction window (30-day all-cause mortality from first contact with health care providers) and a variable-length observation window that expands as clinical data accumulates The predicted outcome is anchored at trajectory start; consequently, the residual prediction window decreases as the trajectory progresses, though the outcome definition itself remains fixed. We use the term “dynamic” throughout this work to refer to this sequential updating of predictions with accumulating data, rather than to a time-varying outcome definition. This framing has direct implications for evaluation, as described below.

Dynamic risk prediction performance was evaluated by masking sequences continuously at each step, equivalent to binning frequencies, to plot AUROC and AUPRC as a function of time. Two complementary evaluation strategies were used to characterize model performance under different assumptions about the evaluable population: population-level evaluation and active-cohort evaluation.

For population-level evaluation, all holdout patients were retained at every evaluation time point regardless of trajectory length. For patients whose hospital trajectory ended before a given evaluation horizon (due to death or discharge), predictions were generated using all available data up to their end point. This approach maintains a fixed cohort across time points, enabling direct comparison of discriminative performance over time without compositional shifts in the denominator population. However, at later evaluation horizons, predictions for short-trajectory patients reflect less temporal information than the horizon implies, and the correlation between trajectory length and outcome introduces informative censoring.

For active-cohort evaluation, each evaluation time point *t* includes only patients with trajectories extending beyond *t*. This ensures that every evaluated prediction is informed by data spanning the full horizon, yielding a clinically interpretable assessment: given a patient still under observation at time *t*, how well does the model discriminate? The trade-off is survivorship bias since the cohort at later time points is progressively enriched for longer-stay patients and decreasing sample size, which widens CI. Sample sizes are reported at each time point for the active-cohort analysis. We present both strategies jointly to allow performance assessment under fixed-population and conditional-on-observation assumptions, respectively.

Additionally, we evaluated the model’s ability to identify high-risk patients using a percentile-based ranking approach. At each time point, predictions were ranked, and the top 5%, 10%, 15%, 20%, and 25% were classified as positive. This approach relies solely on the model’s discrimination (rank-ordering of patients) rather than on absolute predicted probabilities, making it robust to miscalibration. Sensitivity was calculated at each percentile threshold and plotted over time with CIs, illustrating the trade-off between case capture and tolerance to false positives across clinically relevant operating points.

To further assess the reliability of the model’s probability estimates, and following recommendations for comprehensive evaluation of predictive AI models in medicine [21], we created calibration plots, tested for optimal post hoc calibration method through expected calibration error (ECE) metric, conducted post calibration net benefit analysis and calculated the Brier score [22] at multiple time frames using active-cohort evaluation. Furthermore, we optimized classification thresholds using the *F_β_* score, allowing us to balance precision and sensitivity according to the specific applications of our model. We applied *β*=5 for an analysis prioritizing sensitivity and *β*=1 for the harmonic mean between the 2 metrics. These optimized thresholds were then used to construct confusion matrices, which were used for calculating sensitivity, specificity, and positive predictive value (PPV) at a specific threshold.

**Comparison with trauma risk scores**

To contextualize the model’s discriminative performance, we benchmarked against RTS and TRISS. RTS was computed from the first recorded GCS, systolic blood pressure, and respiratory rate using the standard coded-value formulation with Champion coefficients [5]. TRISS was computed from RTS, ISS, age (dichotomized at 55 years), and injury mechanism (blunt vs penetrating) using the Major Trauma Outcome Study logistic regression coefficients. Injury mechanism was determined from Abbreviated Injury Scale region codes.

At each evaluation time point, the model and each score were evaluated on identical patient subsets, restricted to patients with active trajectories for whom the respective score was computable. Coverage varied across scores due to differing input requirements; patients with missing score components were excluded from the respective paired comparison but retained in the model’s primary evaluation. As the traditional scores are time-invariant, computed once from initial presentation data, divergence from the model’s trajectory over time reflects the incremental value of accumulating longitudinal information. Statistical significance of AUROC differences was assessed using paired DeLong tests [23] at each time point, with the Benjamini-Hochberg procedure [24] applied to control the false discovery rate (FDR) at 5%.

### Model Behavior Analysis

To interpret the model’s predictions, we used Shapley additive explanations (SHAP) [25] using the GradientExplainer algorithm, which approximates Shapley values via expected gradients and is well-suited to deep learning architectures [26]. SHA*P* values were computed separately for each input modality: continuous time-series channels, categorical time, static categorical features, and static continuous features.

For categorical time series, we computed SHA*P* values on the raw multihot representation rather than on pre-embedded vectors, yielding per-category attributions that identify which specific medications or procedures contribute to the prediction at each timestep. Static categorical features were pre-embedded before SHAP computation, with attributions averaged across embedding dimensions to produce a single importance score per feature. A background dataset of 1000 randomly sampled training patients served as the reference distribution for the GradientExplainer.

SHA*P* values beyond each patient’s actual trajectory length were zeroed to prevent attribution of importance to padding positions. We computed mean absolute SHA*P* values across holdout patients to identify globally important features and their temporal dynamics. For all time-series modalities, per-measurement importance was computed by averaging absolute SHA*P* values across measured (nonzero) positions only, ensuring comparable density-normalized importance estimates across channels with different sparsity profiles. For temporal analysis, we additionally performed time frame–specific SHAP computation at progressively later clinical time points, censoring input data beyond each time point and recomputing SHA*P* values to assess how feature importance evolves as more clinical information becomes available.

## Results

### Overview

The study included 9496 trauma cases with a mean patient age of 42.2 (SD 22.2) years; 66.4% (n=6308) were male. The mean ECI was 0.7 (SD 3.2), the ISS mean was 6.6 (SD 10.6), and the GCS median was 15.0 (IQR 13.0-15.0). The 30-day mortality rate was 3.92% (372/9496). Sequential data encompassed 8,357,172 vital sign measurements, 2,424,172 medication administrations, and 3,294,268 laboratory test results over a mean patient trajectory of 7.6 (SD 21.5) days. Patient characteristics are available in Table 1.

**Table 1:** Patient characteristics.

Characteristic		Grouped by 30-day all-cause mortality						
		Overall	Survived	Deceased	Missing, n		*P* value	
Patients, n (%)		9496 (100)	9124 (96.08)	372 (3.92)	0		N/A^a^	
Age (years), mean (SD)		42.2 (22.2)	41.2 (21.8)	66.8 (19.9)	0		<.001^b^	
**Sex, n (%)**								.62^c^
	Female	3188 (33.6)	3068 (33.6)	120 (32.3)	0			
	Male	6308 (66.4)	6056 (66.4)	252 (67.7)	0			
Trajectory duration (days), mean (SD)		7.6 (21.5)	7.7 (21.9)	5.4 (6.1)	0		<.001^b^	
Elixhauser Comorbidity Index, mean (SD)		0.7 (3.2)	0.6 (3.0)	3.6 (5.8)	0		<.001^b^	
Height (cm), mean (SD)		168.8 (25.0)	168.6 (25.4)	173.0 (13.0)	3895		<.001^b^	
Weight (kg), mean (SD)		72.2 (23.7)	71.9 (24.0)	76.9 (18.5)	3223		<.001^b^	
Injury Severity Score, mean (SD)		6.6 (10.6)	6.0 (9.5)	19.3 (20.8)	3941		<.001^b^	
Glasgow Coma Score, median (IQR)		15.0 (13.0-15.0)	15.0 (13.0-15.0)	3.0 (3.0-6.0)	116		<.001^d^	
EWS^e^ score, mean (SD)		3.1 (2.8)	3.0 (2.7)	8.1 (3.8)	3840		<.001^b^	
Heart rate (aggregated mean; bpm), mean (SD)		85.9 (16.3)	85.8 (16.1)	89.1 (20.6)	31		.003^b^	
Respiratory rate (aggregated mean; per minute), median (IQR)		16.4 (15.4-18.0)	16.3 (15.4-18.0)	16.7 (14.0-19.4)	1708		.84^d^	
Systolic blood pressure (aggregated mean; mmHg), mean (SD)		126.1 (19.4)	126.3 (19.2)	123.1 (24.5)	47		.02^b^	
Oxygen saturation (aggregated mean; %), median (IQR)		97.5 (96.1-98.5)	97.5 (96.2-98.6)	95.5 (92.1-97.2)	32		<.001^d^	
Temperature (aggregated mean; °C), median (IQR)		36.9 (36.6-37.2)	36.9 (36.6-37.2)	37.0 (36.4-37.4)	2495		.23^d^	
Anesthetics (count of prescriptions), mean (minimum-maximum)		3.9 (0.0-199.0)	3.8 (0.0-199.0)	6.4 (0.0-70.0)	0		<.001^b^	
Antibiotics (count of prescriptions), mean (minimum-maximum)		12.8 (0.0-882.0)	12.7 (0.0-882.0)	15.0 (0.0-152.0)	0		.07^b^	
Antithrombotic agents (count of prescriptions), mean (minimum-maximum)		5.5 (0.0-601.0)	5.6 (0.0-601.0)	3.5 (0.0-41.0)	0		<.001^b^	
Blood products (count of prescriptions), mean (minimum-maximum)		0.2 (0.0-27.0)	0.2 (0.0-21.0)	0.6 (0.0-27.0)	0		<.001^b^	
Cardiovascular drugs (count of prescriptions), mean (minimum-maximum)		5.2 (0.0-839.0)	5.1 (0.0-839.0)	7.9 (0.0-175.0)	0		<.001^b^	
Diuretics (count of prescriptions), mean (minimum-maximum)		2.2 (0.0-496.0)	2.1 (0.0-496.0)	5.1 (0.0-83.0)	0		<.001^b^	
Hemostatics (count of prescriptions), mean (minimum-maximum)		0.4 (0.0-94.0)	0.4 (0.0-94.0)	1.1 (0.0-22.0)	0		<.001^b^	
Infusion (count of prescriptions), mean (minimum-maximum)		4.4 (0.0-584.0)	4.2 (0.0-584.0)	7.9 (0.0-71.0)	0		<.001^b^	
Insulin (count of prescriptions), mean (minimum-maximum)		1.5 (0.0-627.0)	1.4 (0.0-627.0)	3.5 (0.0-74.0)	0		<.001^b^	
Local anesthetics (count of prescriptions), mean (minimum-maximum)		0.6 (0.0-57.0)	0.6 (0.0-57.0)	0.5 (0.0-20.0)	0		.05^b^	
Neurological drugs (count of prescriptions), mean (minimum-maximum)		10.9 (0.0-903.0)	11.1 (0.0-903.0)	6.1 (0.0-84.0)	0		<.001^b^	
Opioids (count of prescriptions), mean (minimum-maximum)		13.0 (0.0-617.0)	13.1 (0.0-617.0)	9.4 (0.0-86.0)	0		<.001^b^	
Base excess (mmol/L), mean (SD)		1.3 (4.0)	1.4 (3.7)	0.8 (6.8)	4074		.13^b^	
Lactate (mmol/L), median (IQR)		2.2 (1.6-3.2)	2.2 (1.5-3.1)	3.9 (2.3-8.4)	2269		<.001^d^	
Hemoglobin (mmol/L), mean (SD)		8.7 (1.0)	8.7 (1.0)	8.0 (1.4)	711		<.001^b^	
Leukocyte count (10^9^/L), median (IQR)		10.3 (7.7-14.2)	10.1 (7.6-13.9)	15.4 (11.1-20.6)	811		<.001^d^	
TEG^f^ reaction time (minute), median (IQR)		5.3 (4.7-6.3)	5.3 (4.6-6.2)	6.0 (4.8-7.5)	8279		<.001^d^	
TEG maximum amplitude (mm), mean (SD)		65.4 (6.9)	65.5 (6.7)	64.6 (8.5)	8279		.30^b^	
TEG fibrinolysis (LY30^g^; %), median (IQR)		0.3 (0.0-1.0)	0.3 (0.0-1.1)	0.0 (0.0-0.6)	8393		<.001^d^	
**A: Airway, n (%)**						1165		<.001^c^
	Clear	7970 (95.7)	7786 (97.1)	184 (58.8)				
	Threatened	327 (3.9)	226 (2.8)	101 (32.3)				
	Blocked	34 (0.4)	6 (0.1)	28 (8.9)				
**B: Breathing, n (%)**						1151		<.001^c^
	Normal	7012 (84.0)	6891 (85.8)	121 (38.7)				
	Lightly affected	1029 (12.3)	952 (11.9)	77 (24.6)				
	Very affected	236 (2.8)	175 (2.2)	61 (19.5)				
	Respiratory arrest (ceased)	68 (0.8)	14 (0.2)	54 (17.3)				
**C: Circulation, n (%)**						1158		<.001^c^
	Normal	6996 (83.9)	6874 (85.6)	122 (39.1)				
	Lightly affected	1018 (12.2)	931 (11.6)	87 (27.9)				
	Very affected	255 (3.1)	205 (2.6)	50 (16.0)				
	Cardiac arrest	69 (0.8)	16 (0.2)	53 (17.0)				
**D: Disability, n (%)**						1172		<.001^c^
	Awake	6703 (80.5)	6613 (82.5)	90 (29.0)				
	Reduced awareness	1237 (14.9)	1161 (14.5)	76 (24.5)				
	Unconscious	384 (4.6)	240 (3.0)	144 (46.5)				
**Level 1 trauma bay visit, n (%)**						0		<.001^c^
	No	4866 (51.2)	4774 (52.3)	92 (24.7)				
	Yes	4630 (48.8)	4350 (47.7)	280 (75.3)				
**Operating room visit, n (%)**						0		<.001^c^
	No	7228 (76.1)	6974 (76.4)	254 (68.3)				
	Yes	2268 (23.9)	2150 (23.6)	118 (31.7)				
**ICU admission, n (%)**						0		<.001^c^
	No	7902 (83.2)	7704 (84.4)	198 (53.2)				
	Yes	1594 (16.8)	1420 (15.6)	174 (46.8)				
**Ward admission, n (%)**						0		.70^c^
	No	4337 (45.7)	4163 (45.6)	174 (46.8)				
	Yes	5159 (54.3)	4961 (54.4)	198 (53.2)				

^a^N/A: not applicable.

^b^Welch *t* test.

^c^Chi-square test.

^d^Kruskal-Wallis test.

^e^EWS: Early Warning Score.

^f^TEG: thrombelastography.

^g^LY30: Lysis-index 30.

The training process used 7667 (80%) trauma cases, and 1829 (20%) cases were held out for independent validation. The temporally split holdout cohort showed notable distributional differences, most prominently a reduction in level 1 trauma bay contact, shorter trajectory durations, and lower rates of operating room visits and ward admissions, compared to the development cohort, while outcome prevalence remained comparable (Table S5 in Multimedia Appendix 1).

### Performance

Predicting 30-day mortality for the holdout dataset using full-length sequences, the model achieved an AUROC of 0.962 (95% CI 0.930-0.994) and an AUPRC of 0.655 (95% CI 0.545-0.765). ROC and PR curves at selected trajectory time points are presented in Figure S3 in Multimedia Appendix 1.

The model demonstrated sensitivity ranging from approximately 0.49 to 0.96 within the first 12 hours, with 5 to 25 top percent predictions marked as positives. Sensitivities across the trajectory for top 5 to 25 percent high-risk patients are presented in Figure S4 in Multimedia Appendix 1.

The model performance varied depending on prediction time in the patient trajectory. For population-level evaluation, the first 12 hours AUROC was 0.905-0.936 and AUPRC 0.388-0.461, from 12 to 72 hours AUROC was 0.936-0.954 and AUPRC 0.461-0.557, from 72 hours to 7 days AUROC was 0.954-0.957 and AUPRC 0.557-0.601, from 7 days to 30 days AUROC was 0.957-0.962 and AUPRC 0.601-0.655. For active-cohort evaluation, the first 12 hours AUROC was 0.887-0.926 and AUPRC 0.375-0.452, from 12 to 72 hours AUROC was 0.874-0.919 and AUPRC 0.375-0.409, from 72 hours to 7 days AUROC was 0.919-0.921 and AUPRC 0.316-0.409, from 7 days to 30 days AUROC was 0.905-0.921 and AUPRC 0.316-0.350. Both evaluation strategies with CIs and active cohort composition are presented in Figure 3 (presented with CIs in Table S6 in Multimedia Appendix 1).

**Figure 3:**
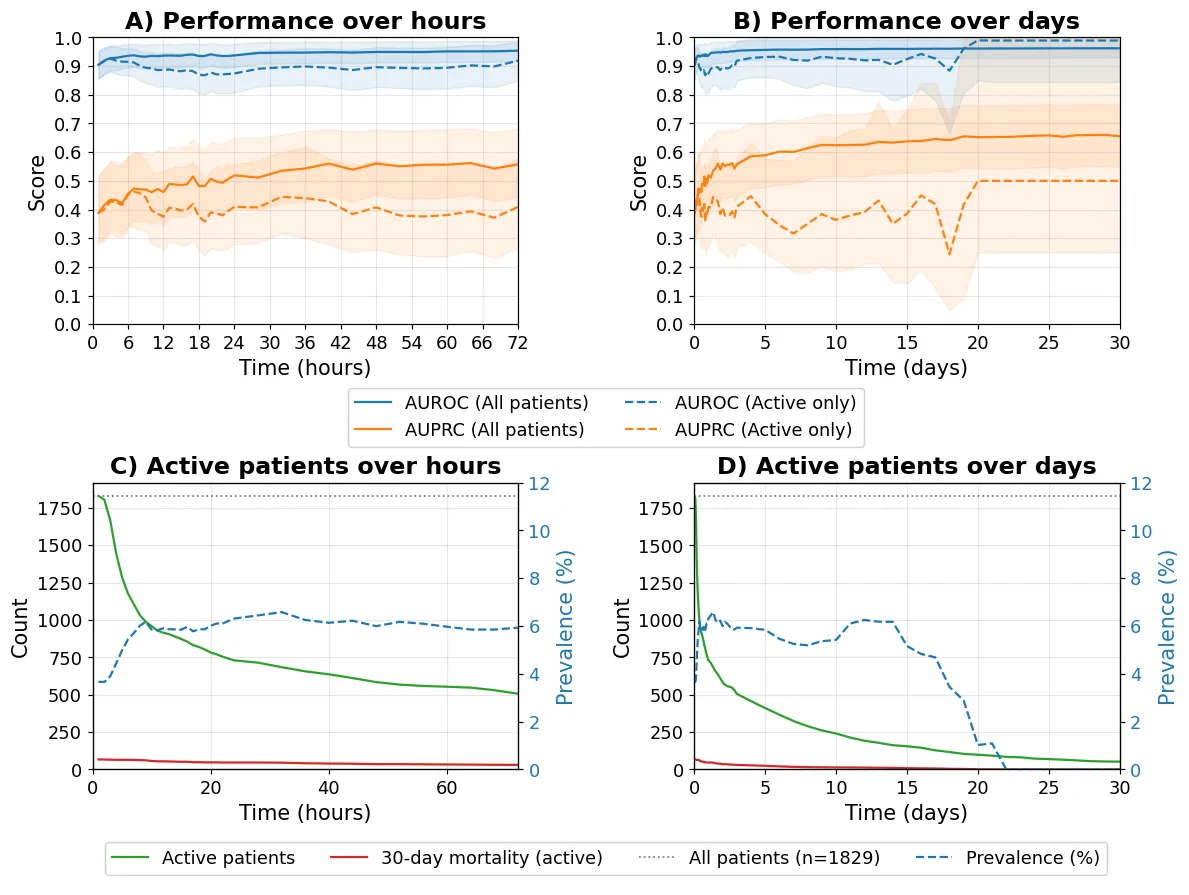
Dynamic evaluation of model performance and active cohort composition, starting at first prehospital data point registration. (A) Area under the receiver operating characteristic curve (AUROC) and area under the precision-recall curve (AUPRC) over the first 72 hours for population-level (all patients: solid lines) and active-cohort (dashed lines) evaluation strategies, with 95% CIs. (B) AUROC and AUPRC over 30 days using the same evaluation strategies. (C) Number of active patients (left axis) and 30-day mortality prevalence among the active cohort (right axis, %) over the first 72 hours. The dotted line indicates the total holdout population (n=1829). (D) Active patients and prevalence over 30 days.

### Calibration and Clinical Use

The raw model outputs exhibited overconfidence across evaluation time points. Post hoc calibration was performed using isotonic regression and Platt scaling, each fitted per time point on the development dataset and applied to the holdout predictions. Isotonic regression yielded lower ECE and Brier scores than Platt scaling beyond 1 hour and was selected for subsequent analyses (Figure S5 in Multimedia Appendix 1). Following isotonic calibration, low-probability bins tracked the diagonal more closely across time points, though reliability estimates became unstable at later time points (7 days and 14 days) due to sparsely populated calibration bins in the shrinking active cohort. Post hoc isotonic calibration reduced Brier scores from 0.037 to 0.027 at 1 hour and from 0.062 to 0.049 at 14 days. Reliability diagrams with Brier scores at selected time points are presented in Figure S6 in Multimedia Appendix 1.

Decision curve analysis using isotonic-calibrated outputs demonstrated positive net benefit over the “treat all” and “treat none” strategies across a range of clinically relevant threshold probabilities at all evaluated time points (1 hour, 6 hours, 12 hours, 3 days, 7 days, and 14 days; Figure 4). Net benefit was highest at low threshold probabilities and decreased as the threshold increased. At 7 and 14 days, the net benefit curves exhibited greater variability, consistent with the reduced active cohort size at later time points.

**Figure 4:**
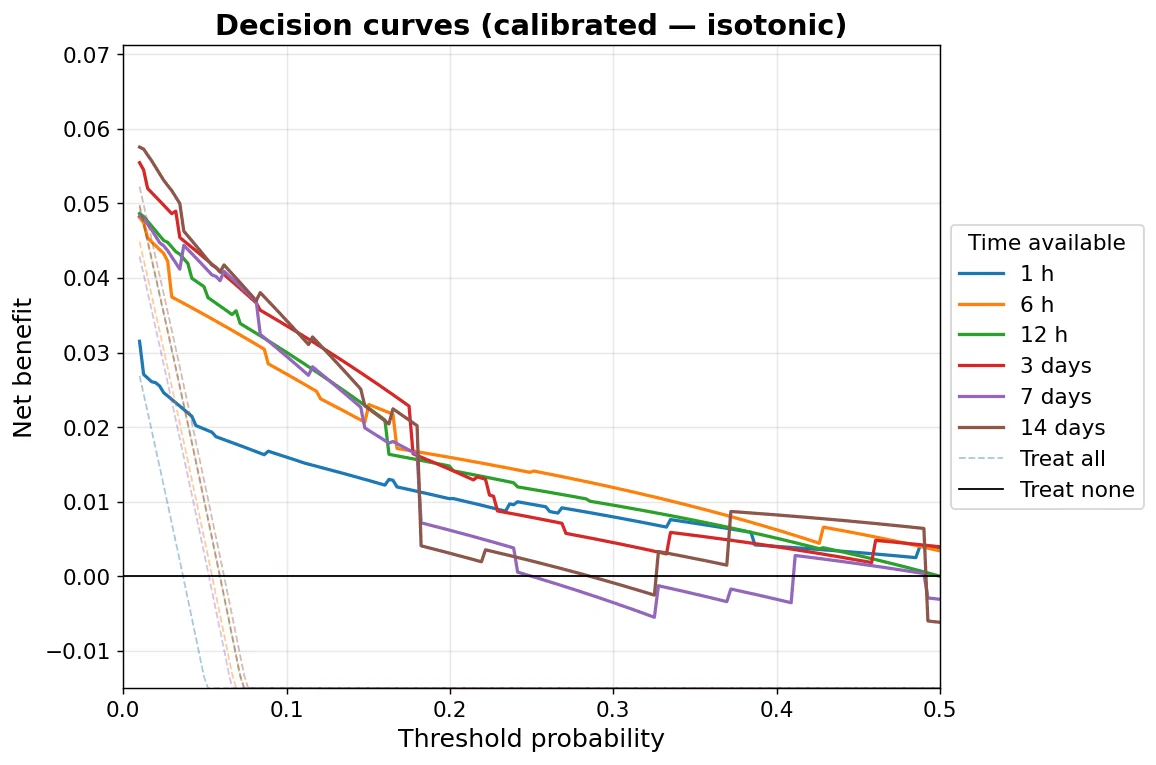
Decision curve analysis showing net benefit of the model at selected evaluation time points (1 hour, 6 hours, 12 hours, 3 days, 7 days, and 14 days) across a range of threshold probabilities. Model outputs were calibrated using isotonic regression fitted per time point on the development dataset and applied to the active holdout set. Dashed lines represent the “treat all” strategy at each time point. The solid black line at zero represents the “treat none” strategy.

Predicted probability distributions stratified by outcome are presented in Figure S7 in Multimedia Appendix 1. Across all time points, the distributions for deceased patients were concentrated at higher predicted probabilities than for survivors.

*F_β_* optimized thresholds were calculated from internal cross-validation with the final hyperparameters. Using *β* of 5 (t=0.040), the model achieved 84% sensitivity and 93% specificity (Figure S8 in Multimedia Appendix 1), with a PPV of 30%. In absolute numbers, this means identifying 56 of 67 deaths while wrongly labeling 128 cases. Using the harmonic mean of precision and sensitivity (*β*=1, t=0.500), the model achieved 46% sensitivity and 99% specificity (Figure S9 in Multimedia Appendix 1), with a PPV of 76%. In absolute numbers, this identifies 31 of 67 deaths with 10 false positives.

### Comparison With Trauma Scores

The comparisons were conducted on holdout patients eligible for each respective score, restricted to those with the registrations required for trauma score calculation, resulting in varying cohort sizes across time points and scores. TRISS requires ISS, which in our implementation enters the model sequentially as diagnosis codes are registered, but was made available to TRISS at the earliest available registration regardless of evaluation time point to maximize the comparable cohort.

Compared with RTS (n=1401), the model achieved a mean AUROC advantage of +0.297, reaching significance (FDR-corrected *P*<.05) at 50 of 54 evaluated time points (Figure 5).

**Figure 5:**
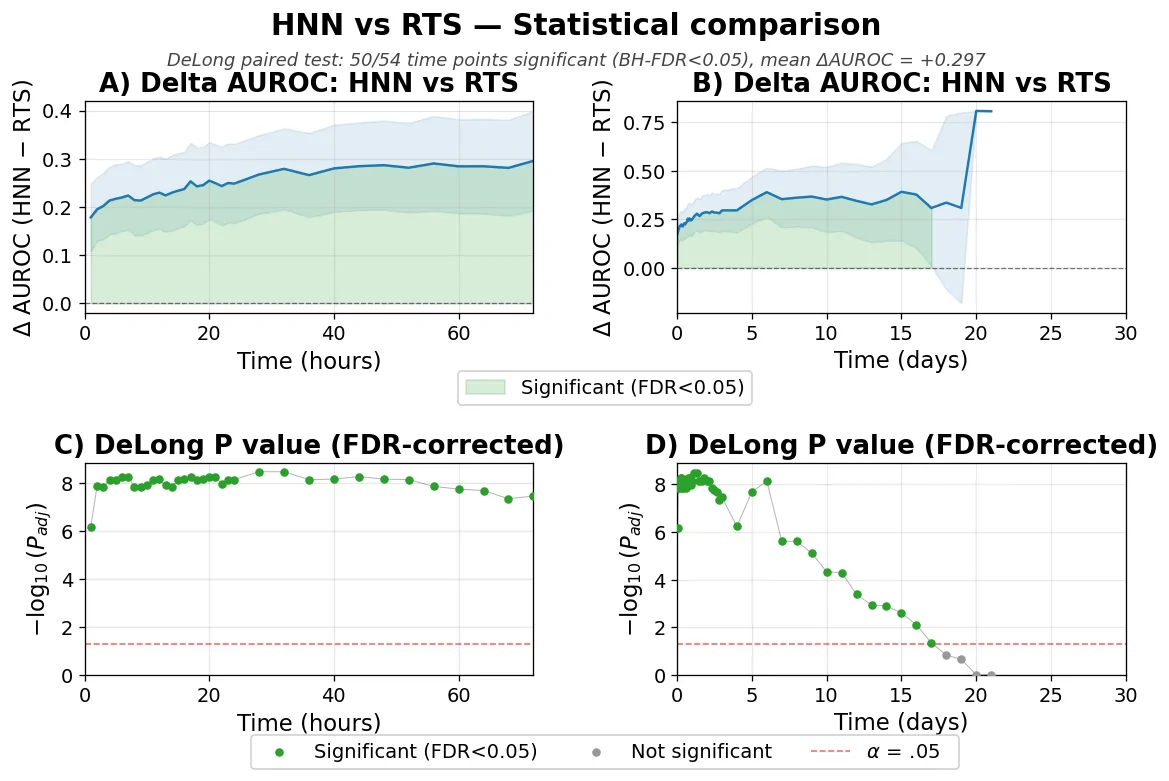
Statistical comparison of area under the receiver operating characteristic curve (AUROC) between the hybrid neural network (HNN) and Revised Trauma Score (RTS) using paired DeLong tests with Benjamini-Hochberg false discovery rate (FDR) correction. (A) ΔAUROC (HNN minus RTS) over the first 72 hours with 95% CIs. Green shading indicates time points with FDR-corrected significance (*P*<.05). (B) ΔAUROC over 30 days. (C) FDR-corrected *P* values displayed as –log₁₀(p_adj) over the first 72 hours. Green points indicate significance below the α=0.05 threshold (dashed red line); gray points indicate nonsignificant comparisons. (D) FDR-corrected *P* values over 30 days.

Compared with TRISS (n=878), the model achieved a mean AUROC advantage of +0.169, with significance at 46 of 54 time points (Figure 6). For both comparisons, significance was lost at later time points (beyond approximately 15 days). AUROC and AUPRC across time for each comparison are presented in Figures S10 and S11 in Multimedia Appendix 1.

**Figure 6:**
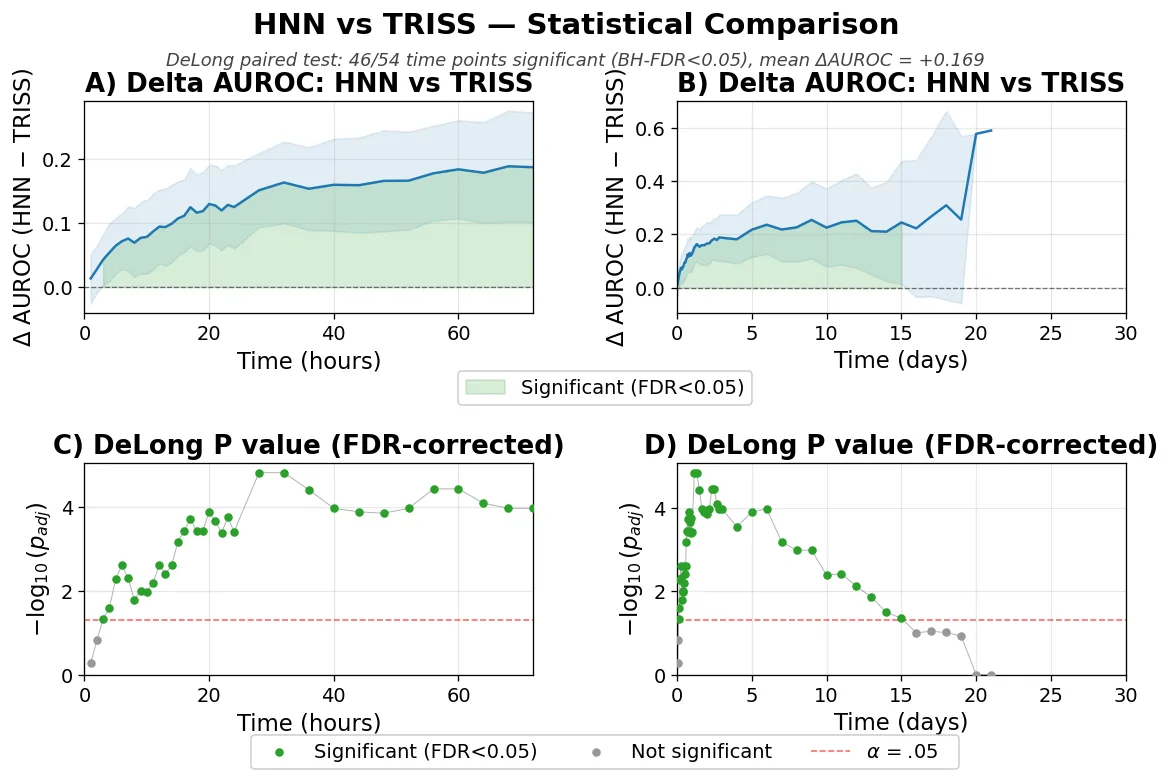
Statistical comparison of area under the receiver operating characteristic curve (AUROC) between the hybrid neural network (HNN) and Trauma and Injury Severity Score (TRISS) using paired DeLong tests with Benjamini-Hochberg false discovery rate (FDR) correction. (A) ΔAUROC (HNN minus TRISS) over the first 72 hours with 95% CIs. Green shading indicates time points with FDR-corrected significance (*P*<.05). (B) ΔAUROC over 30 days. (C) FDR-corrected *P* values displayed as –log₁₀(p_adj) over the first 72 hours. Green points indicate significance below the α=0.05 threshold (dashed red line); gray points indicate nonsignificant comparisons. (D) FDR-corrected *P* values over 30 days.

### Model Behavior

SHAP analysis at the cohort level revealed that static patient features contributed most to model predictions. Age was consistently among the highest-contributing features across all evaluated time frames (mean |SHAP| 0.171 at 1 hour, increasing to 0.465 at 14 days; Figure 7). ECI was the second-highest static continuous contributor, surpassing age at intermediate time frames (0.530 vs 0.455 at 7 days) before declining at later time points. Among static categorical features, the prehospital disability (D) assessment was the most important (0.136 at 1 hour, 0.445 at 7 days), followed by breathing (B) assessment and first hospital of admission.

**Figure 7:**
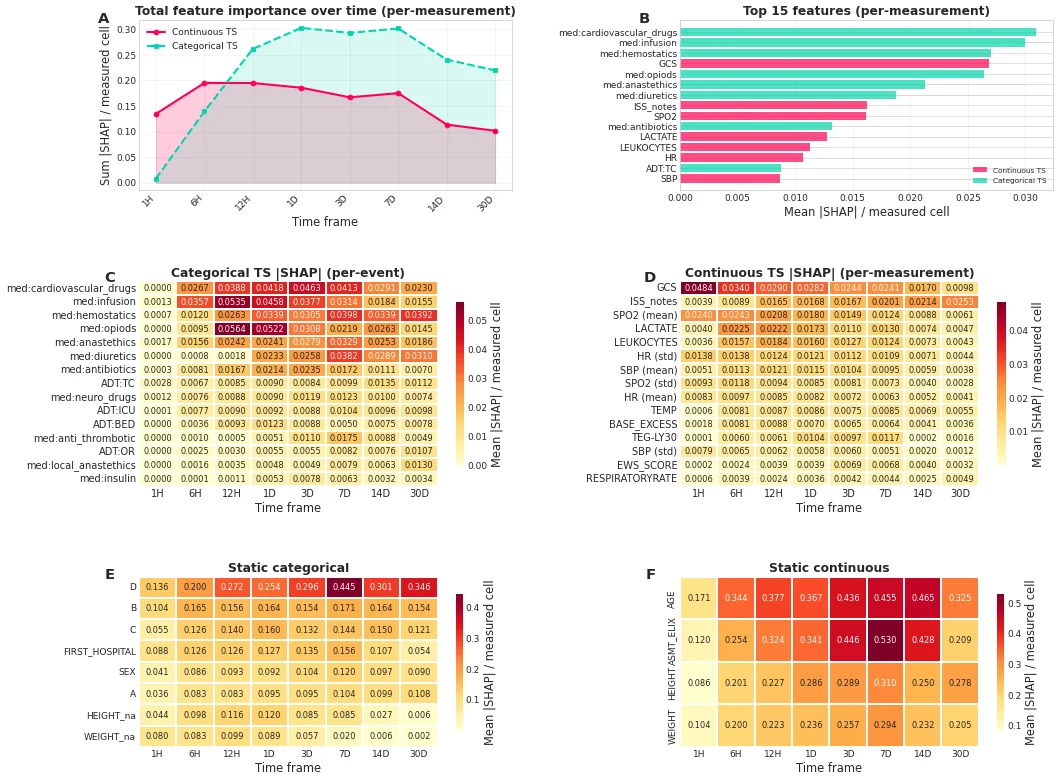
Cohort-level Shapley additive explanations (SHAP) analysis of feature importance across evaluation time frames using active-cohort patients computed using GradientExplainer with a background dataset of 1000 training patients. (A) Sum absolute SHA*P* value per measured cell for continuous and categorical time series (TS) features over time. (B) Top 15 time series features ranked by mean absolute SHAP per measured cell across the full trajectory, showing both continuous and categorical channels. (C) Mean absolute SHA*P* values per measured cell for categorical time series features across time frames. (D) Mean absolute SHAP per measured cell for continuous time series channels across time frames. (E) Mean absolute SHA*P* values for static categorical features across time frames. (F) Mean absolute SHA*P* values for static continuous features across time frames.

Among time-series features, per-measurement importance was shared between continuous channels and categorical (medication and ADT) channels (Figures 7A-7D). Among continuous time-series channels, GCS had the highest per-measurement importance at early time points (0.0484 at 1 hour), followed by mean SpO_2_ (0.0240) and heart rate variability (SD 0.0138). GCS had continuously high importance throughout available time frames. Lactate demonstrated high importance at 6 to 24 hours with decline, thereafter, followed by leukocytes displaying a similar pattern. ISS derived from clinical notes showed increasing contribution as the time frame expanded, becoming the most important continuous channel by 14 days (0.0214). Per-measurement importance of continuous time-series features decreased over time as the trajectory progressed (Figure 7A). Among categorical time-series channels, several medication categories dominated, with opioids and infusion fluids having the highest per-measurement importance at 12 hours (0.0564 and 0.0535, respectively), comparable in magnitude to the top continuous channels at the same time point (GCS: 0.0290). Cardiovascular drugs peaked at 3 days (0.0463), while hemostatics showed sustained importance across the full trajectory, peaking at 7 days (0.0398). On a per-measurement basis, the highest-contributing medication categories were comparable in magnitude to the top continuous channels.

The relative contribution of static features increased over time while per-measurement importance of temporal features decreased. Full cohort-level SHA*P* values across time frames are presented in Table S7 in Multimedia Appendix 1.

## Discussion

### Principal Findings

This study focused on constructing trajectories for patients with trauma automatically from available EHRs for dynamic risk prediction of 30-day mortality. We found that using a hybrid neural network for predicting 30-day mortality achieved an AUROC of 0.962 and an AUPRC of 0.655 on a holdout dataset using full-length sequences. Given the 30-day mortality rate of 3.92% (372/9496), the AUPRC substantially exceeds the baseline prevalence, indicating strong discriminative performance despite marked class imbalance. Raw model outputs exhibited overconfidence, which post hoc isotonic calibration substantially reduced, improving alignment with observed outcomes particularly at lower predicted probabilities, which is the range most relevant to ruling out deterioration in clinically stable patients.

The dynamic evaluation of performance demonstrated varying predictive performance over time, with cumulative discrimination improving as trajectories progress. To characterize this under different assumptions, we used 2 complementary evaluation strategies: population-level evaluation, retaining all patients at every time point, and active-cohort evaluation, restricted to patients with trajectories extending beyond each time point. The patient trajectory duration followed a positively skewed distribution with most patients having less than 7 days duration (Figure 3), resulting in decreasing active cohort sizes and widening CIs at later time points, presenting an important aspect for interpreting the presented results. Due to the temporal holdout split, the last active patient died near day 21, making evaluation beyond this point unreliable or impossible.

### Model Behavior

SHAP analysis at the cohort level revealed that static patient features, particularly age and ECI, contributed most to model predictions across the trajectory. Among time-series features, GCS, ISS, SpO_2_, and heart rate variability were the most important continuous channels. Categorical time-series features were dominated by medication categories, with cardiovascular drugs, infusion fluids, and hemostatics ranking among the highest per-measurement contributors. The high importance of medication features is clinically plausible, as the initiation and intensity of pharmacological intervention directly reflects the treating clinician’s assessment of patient severity, though the observational design precludes distinguishing prognostic signal from treatment-outcome confounding.

Early, high SHA*P* values for vitals and GCS align with these being the primary data available during the prehospital phase, where prehospital ABCD assessments provided complementary information, with the disability (D) assessment the most important static categorical feature spanning across all time frames.

Temporal analysis of feature importance showed that the relative contribution of static features increased at later time points while per-measurement importance of individual temporal features generally decreased, though total categorical time-series importance remained elevated through intermediate time frames. These findings support the clinical plausibility of the model’s learned representations and provide transparency into which data streams drive risk estimation at different phases of the trauma trajectory.

### Clinical Use and Implications

In most clinical applications, the model output would be presented to clinicians as a calibrated probability with reference levels rather than a binary classification, functioning as a continuously updated severity index analogous to existing trauma scores but with sequential updating capability presenting risk trend over time. This approach avoids the information loss inherent in dichotomizing a continuous risk estimate and allows clinicians to interpret the output in the context of their own decision framework. However, certain applications require binary classification, such as selecting cases for quality-of-care review or triggering automated deterioration alerts. For screening applications that prioritize sensitivity, we optimized the threshold using *F_β_* (*β*=5) on the validation dataset, achieving 84% sensitivity and 93% specificity, identifying 56 of 67 deaths while generating 128 false positives. For settings where actionable precision is paramount, optimizing by *F*_1_ yielded 76% PPV, identifying 31 of 67 deaths with only 10 false positives. The acceptable trade-off between sensitivity and false-positive burden will vary by use case and time point, and threshold selection should be guided by institutional priorities.

Deploying sequential risk prediction in clinical practice introduces risks that must be addressed alongside discriminative performance. A primary concern is alert fatigue: if the model generates frequent or nonactionable alerts, clinicians may habituate to the output and disregard clinically meaningful signals. Because the model generates a prediction at every evaluation time point, the total alert volume per patient substantially exceeds that of a single-admission score. The threshold analyses presented above were conducted on full-sequence predictions, where each patient contributes a single prediction based on their complete trajectory, and therefore represent an upper bound on achievable precision. While the *F*_1_-optimized threshold yielded only 10 false positives across the holdout set under these conditions, per–time point prediction with incomplete trajectories, particularly in the early phase where model uncertainty is highest, would likely produce a higher operational false-positive burden. A parallel concern applies at the late-trajectory tail, where sparse event density limits the stability of calibrated probabilities and warrants additional caution in any alerting logic that depends on probability magnitude rather than on relative risk ranking. Characterizing the per–time point alert rate under sequential deployment is an important direction for future implementation research. Strategies for mitigating alert fatigue include restricting alert triggers to threshold crossings or sustained risk elevations rather than displaying continuous output, suppressing repeated alerts for the same patient within a defined time window, and tailoring alert thresholds to clinical context (eg, lower thresholds in the trauma bay or higher thresholds on the ward).

The risk of overreliance, where clinicians defer excessively to model output at the expense of independent clinical judgment, is equally relevant. The model is intended to augment, not replace, clinical decision-making. Model outputs should be interpreted as one input among many for clinical decision-making, particularly in the early trajectory where limited data yields wider uncertainty. Institutional safeguards such as structured training on model limitations, transparent communication of prediction uncertainty, and periodic auditing of decision patterns following model deployment are essential to ensure that the tool supports rather than replaces clinical reasoning. Prospective evaluation of these integration safeguards will form a core component of future implementation studies.

The sequential prediction framework supports several clinical applications across the trauma care continuum. First, continuously updated risk estimates can serve as an urgency signal in the trauma bay, where the initial clinical picture is often incomplete and rapidly evolving. A rising predicted mortality in the first hours may prompt earlier escalation to definitive surgical intervention or activation of additional resources. Second, the model output can function as a structured communication tool during transitions between treatment phases (eg, from the trauma bay to the operating room, or from the operating room to the ICU), providing receiving teams with an objective, data-driven summary of trajectory severity that complements verbal handover. Third, in the operative setting, real-time risk estimates may inform treatment strategy decisions, such as the choice between damage-control and definitive surgery. Fourth, in the inpatient setting, continued risk assessment may serve as a patient deterioration alert or as a tool for prioritizing patients during ward rounds, enabling clinicians to allocate attention to those with rising or persistently elevated risk trajectories.

It is important to note that although the model incorporates prehospital data, it is not intended for prehospital decision-making in the current scope. Patients declared dead on scene were excluded from the study cohort, and the model requires data from the hospital trajectory to generate meaningful sequential predictions. The target population is therefore patients who survive to in-hospital trauma team activation, with prehospital data serving as early contextual input that enriches the initial prediction rather than as a stand-alone prehospital triage tool.

### Comparison With Prior Work

In this study, we directly compared the model performance to the 2 trauma scores: RTS and TRISS. Because each score requires specific registrations for calculation, comparisons were restricted to eligible patients at each time point, resulting in varying cohort sizes across time points and scores.

Several additional caveats apply. First, TRISS requires ISS, which in our model enters sequentially as registrations become available. To maximize the comparable cohort, we provided ISS to TRISS from the earliest registration regardless of time point, effectively giving TRISS an informational advantage. Second, neither trauma score was designed for longitudinal risk prediction: RTS was developed for field triage, and TRISS for benchmarking trauma care quality against the Major Trauma Outcome Study cohort. Despite these advantages afforded to the baseline scores, the hybrid neural network significantly outperformed both across the majority of evaluation time points, with a mean AUROC advantage of +0.297 over RTS and +0.169 over TRISS. After FDR correction, the advantage was significant at 50 of the eligible 54 time points for RTS and 46 of 54 for TRISS. Statistical significance was lost beyond approximately 15 days, where decreasing active-cohort sizes reduced statistical power.

Our model’s performance compares with recent advancements in trauma mortality prediction. The Parkland Trauma Index of Mortality (PTIM) [27] is an ML algorithm predicting 48-hour mortality during the first 72 hours of hospitalization for patients with polytrauma, reporting an AUROC of 0.94 for 516 cases. PTIM uses 23 variables, including vital signs, laboratory values, clinical scores, and demographics. One notable advantage of the PTIM the integration into the EHR system, allowing for real-time, hourly updates of mortality predictions. This feature enhances its clinical use, as demonstrated in a survey-based study where 77% of providers reported that the PTIM assisted in their treatment decisions [28].

Moreover, the Epic Deterioration Index (EDI) [29] also demonstrated impressive results, with an AUROC of 0.98 for predicting in-hospital mortality within 24 hours of admission for 1325 level 1 trauma center patients. Both the PTIM study and the EDI validation study operate on shorter time frames compared to our study, which focuses on 30-day mortality prediction. Moreover, our study included a larger and more heterogeneous population, whereas PTIM only included patients with polytrauma, and the EDI validation included only level 1 trauma center patients. Particularly, these differences limit direct performance comparisons but demonstrate the feasibility of ML-based mortality prediction models in trauma care.

Traditionally, risk estimation often required manual data entry into separate interfaces, with information gathered from the EHR and then entered into a distinct system for risk calculation, such as the Predictive Optimal Trees in Emergency Surgery Risk calculator [30]. In our approach, we have concentrated on input features that can be automatically collected, ensuring that risk predictions can be generated with minimal manual effort and made readily available. While difficult, this strategy reduces the burden on health care providers and enables real-time risk assessment and reduces the potential for human error [31].

### Study Limitations

This study has several limitations that should be considered when interpreting the results.

First, the population consists of patients in a single region in eastern Denmark, thus not fully representing the entire demographic spectrum of patients with trauma and may contain population bias. Moreover, the data might reflect local practices and guidelines, such as in the predefined categories for ABCD assessment. Despite these specific categories being implemented and used nationally, there may be regional differences in how clinicians are instructed to conduct subjective assessments. Within the study period itself, comparison of baseline characteristics between the development and holdout cohorts (Table S5 in Multimedia Appendix 1) revealed notable distributional differences between cohorts, most prominently a considerable reduction in level 1 trauma bay contact, alongside shorter trajectory durations and lower rates of operating room visits, while outcome prevalence remained stable and core injury characteristics including ISS and ECI were comparable. No changes to regional triage protocols were implemented during the study period, and the cause of this shift is unknown. Because the SHAP analysis identifies facility-based features among the contributors to model predictions, this reduction represents a shift in one of the model’s informative inputs, not merely a background cohort characteristic. If the lower rate of level 1 trauma bay contact reflects a genuine change in case mix, the feature retains its prognostic relevance; if it instead reflects unmeasured changes in referral patterns among patients of comparable severity, the learned association between trauma bay contact and mortality risk may not generalize to settings where such patterns have shifted further. Disentangling these mechanisms is not possible from observational data alone. That the model maintained strong discrimination despite this distributional shift provides some evidence of robustness, but prospective validation with contemporaneous cohort definitions is needed to confirm generalizability under continued system-level changes.

If the model is to be used in other countries or regions, retraining with local data is highly advisable [9,32], as trauma systems and underlying patient demography vary substantially even between comparable Western health care systems. Further studies should investigate whether similar modeling approaches yield comparable predictive performance when using different structures of the same information. This research should focus on varied data representations (eg, structured vs unstructured data), alternative categorization schemes, and alternative encoding methods.

Second, the binary classification framework does not account for time-to-event dynamics and introduces evaluation challenges for patients whose trajectories end before a given evaluation time point. We address this through dual evaluation strategies (population-level and active-cohort), but both carry inherent trade-offs: informative censoring in the former and survivorship bias in the latter.

Third, an important consideration in interpreting the sequential evaluation results is the model’s framing structure. The prediction target, 30-day all-cause mortality from first contact with health care providers, remains fixed across all evaluation time points, while the observation window expands as more clinical data becomes available. This means that the observed improvement in discriminative performance over time reflects 2 concurrent effects: the model receiving richer input data, and the effective prediction horizon shrinking as the elapsed time since trauma activation increases. At early time points, the model must predict an outcome up to 30 days in the future with limited information; at later time points, much of the outcome-relevant period has already elapsed. Disentangling these 2 contributions is not possible within this study design and represents a limitation of the fixed–end point evaluation framework. We adopted this framing to support future extension to multiple clinical outcomes that are not naturally suited to time-to-event modeling, such as massive transfusion, mechanical ventilation, and posttrauma complications, for which binary classification provides a more generalizable structure.

Fourth, the exclusion of patients declared dead on scene creates a survivor cohort that does not capture the most extreme end of the severity spectrum. This exclusion is intentional, as the model requires sequential hospital data to generate predictions and is therefore applicable only to patients surviving to hospital admission. However, it limits the model’s ability to learn from the highest-acuity prehospital presentations and should be considered when interpreting early-trajectory predictions.

Fifth, post hoc calibration using isotonic regression was fitted on the training and validation set and applied to the holdout set, which together comprise 372 outcome events. Because isotonic regression is nonparametric, it is susceptible to overfitting in sparse regions of the input distribution, which in our setting correspond to the late-trajectory time points where the active cohort shrinks and both event counts and prediction diversity decline. The reliability diagrams at later time points reflect this instability. In a clinical deployment, overfit calibration at the late tail could translate into misleading probability estimates in precisely the region where the model is already most uncertain, compounding the alarm fatigue and overtriage risks discussed above. Mitigation strategies include restricting calibrated probability presentation to time points with adequate event density, adopting parametric alternatives such as logistic calibration for the late-trajectory region, or periodic recalibration as additional patient trajectories accumulate. Future work with larger validation cohorts would strengthen confidence in calibration and clinical use assessments across the full prediction horizon.

Sixth, the inclusion of therapeutic interventions (including blood transfusions, vasopressors, and anesthetics) as sequential input features introduces the potential for treatment-outcome confounding, commonly referred to as the treatment paradox. The model may learn to associate aggressive interventions with higher mortality risk, reflecting the severity of the underlying condition rather than a causal effect of treatment. We consider this an intentional design choice: in clinical practice, the initiation of aggressive interventions (eg, massive transfusion protocol activation) itself conveys prognostic information, and experienced trauma clinicians interpret such interventions as indicators of severity that shape subsequent management decisions. The model is designed to mirror this clinical reasoning pattern, integrating treatment signals as markers of evolving patient state. SHAP analysis showed that medication-related features, particularly cardiovascular drugs, infusion fluids, and hemostatics, were among the highest-contributing categorical time-series channels on a per-measurement basis, indicating that the model does rely on treatment signals for its predictions. While the clinical plausibility of these associations is high (patients receiving aggressive pharmacological intervention are more severely injured), the observational nature of the training data precludes causal inference, and users should be aware that the model captures associations between treatment patterns and outcomes, not treatment effects. Disentangling the prognostic value of treatment signals from treatment-outcome confounding would require causal inference methods or experimental designs that are beyond the scope of this study.

Seventh, whether the transformer-based architecture offers a performance advantage over simpler model classes (such as gradient-boosted trees or recurrent neural networks) remains an open empirical question. The objective of this study was to demonstrate the feasibility of sequential modeling for trauma risk prediction, not to identify the optimal architecture for this task. The transformer was selected for its capacity to process variable-length multivariate sequences with attention-based weighting, but simpler architectures may achieve comparable discrimination with lower computational cost and greater interpretability. Systematic architecture comparison represents a valuable direction for future work, ideally conducted on a shared benchmark dataset to enable direct comparison across studies.

Eighth, SHA*P* values were calculated on subsets for both background (n=1000) and analysis (n≤500) rather than full cohorts, which may influence importance estimates, particularly given the low outcome prevalence. This was done due to computational limits. Full-cohort SHAP analysis and systematic ablation studies would be needed to confirm the relative feature importance rankings and to determine whether any input modalities could be excluded without performance degradation.

Ninth, the temporal positioning of ISS within the sequential input relies on diagnosis registration dates for *ICD*-derived scores and note last-edited time stamps for free-text extractions. Both are imperfect proxies for the point at which the information was clinically derivable. Last-edited time stamps can postdate this point when notes are amended after initial documentation. This was a deliberate conservative choice against data leakage, with the trade-off that the model may receive the information later than it was truly derivable.

Tenth, this study does not include a subgroup analysis of predictive performance across sociodemographic strata. The descriptive statistics indicate significant differences in age and sex distribution between survivors and decedents, and trauma care delivery is known to differ across demographic groups in ways that can propagate into model behavior. Potential sources of inequity include uneven representation of older patients and women in the training cohort, differential documentation patterns across prehospital services, and outcome label heterogeneity driven by differences in goals-of-care decisions near end of life. Evaluating whether the model achieves comparable discrimination, calibration, and net benefit across age brackets and between sexes is a necessary step before clinical deployment. We have deferred this analysis to the prospective validation protocol under development, where subgroup definitions, sample size requirements, and the fairness criterion (eg, equalized odds or calibration parity) can be prespecified against a defined clinical-use framework rather than tested post hoc on the holdout set. Until that evaluation is completed, model outputs should be interpreted with awareness that subgroup-specific performance has not been established.

Eleventh, the benchmark comparisons against RTS and TRISS were restricted to patients for whom the respective scores were computable at each evaluation time point. RTS requires initial GCS, systolic blood pressure, and respiratory rate, all of which are systematically missing in patients receiving early airway management (including prehospital intubation) and in those with unobtainable vital signs due to hemodynamic collapse. Such patients are, on average, the most severely injured in the cohort. Their exclusion biases the RTS-eligible subcohort toward lower acuity and likely causes the observed performance gap between the hybrid neural network and RTS to underestimate the true advantage among the most critical patients, where both methods would be evaluated on a cohort with more homogeneous baseline risk. TRISS is subject to the same eligibility-driven bias through its dependence on RTS components, compounded by additional ISS and injury-mechanism requirements. The comparative metrics reported here should therefore be interpreted as a lower bound on the model’s advantage over traditional scores in the full trauma cohort.

### Future Directions

While our model demonstrates strong predictive performance, there are several avenues for potential improvement and future research. This study focused on 30-day mortality, where predicting additional outcomes such as massive transfusion, ICU admission with mechanical ventilation, major surgical interventions, posttrauma complications, readmissions, or functional status could provide a more comprehensive risk assessment. Another application of this modeling approach was demonstrated by our research group using a prehospital EHR to predict surgical needs stratified into specialties [33]. Adapting the model to predict several outcomes relevant at different decision-making branching points could significantly impact patient care from the earliest stages of trauma management to hospital discharge.

Incorporating additional data types could also enhance the model’s predictive power. However, careful consideration must be given to avoid data leakage, particularly when including diagnoses and procedures that might be directly related to the outcome. Future studies should explore integrating such information while maintaining the integrity of the prediction task. Another area for improvement lies in exploring different data representations and aggregations. For instance, medications and laboratory results could be grouped or aggregated in various ways to capture more nuanced patterns. These alternative representations might reveal additional insights and improve the model’s performance. Research into optimal grouping strategies for different data types could yield significant advancements in predictive accuracy.

Future studies should further scrutinize subgroup model performance and behavior analysis to identify optimal configurations between predictive performance and clinical use. Next-phase evaluation would assess the effect of our model in the clinic by randomized controlled trials prescribing our model, using well-established quality indicators as outcomes, such as those used in the Trauma Quality Improvement Program by the American College of Surgeons [34]. This would quantify clinical benefits while identifying implementation barriers and care gaps. Our study demonstrates the feasibility and potential of constructing patient trajectories from EHRs for dynamic risk prediction, showcased by the model’s strong performance in predicting 30-day mortality. While there are clear areas for future improvement and expansion, this approach advances toward more data-driven, personalized trauma care. As we continue to refine and expand these models, their integration into clinical practice could support trauma care from prehospital triage to long-term management. While these tools enhance clinical judgment rather than replace it, their integration could improve trauma outcomes through timely, insight-driven decision support.

### Conclusions

This study demonstrates that a transformer-based sequential model trained on EHR data spanning prehospital and in-hospital care can predict 30-day all-cause mortality in patients with trauma with strong discrimination across the care trajectory. The model significantly outperformed established trauma risk scores (RTS and TRISS) at the majority of evaluation time points, with the advantage increasing as longitudinal data accumulated. SHAP-based interpretability analysis indicated that predictions were driven primarily by static patient characteristics, physiological measurements, and treatment patterns, with prehospital clinical assessments providing complementary early information. Post hoc isotonic calibration yielded well-calibrated risk estimates suitable for presentation as a continuous severity index, and decision curve analysis demonstrated positive net benefit over default strategies across a range of clinically relevant threshold probabilities at multiple time frames. These findings support the feasibility of sequential transformer-based modeling for dynamic trauma risk prediction. Future work will focus on prospective evaluation of clinical integration, targeting urgency awareness, interphase communication, operative decision support, and inpatient deterioration detection in the acute trauma setting.

## Data Availability

Due to confidentiality issues, we are not at liberty to share the electronic health record (pre- and in-hospital) data used in this study. Access to Danish health data is granted through the Danish Patient Safety Authority. EHR data can then be obtained by reasonable request through the relevant region. Project source code is available at the following GitHub repository: https://github.com/a-millarch/dynamic-risk-prediction.

## Appendix

Multimedia Appendix 1 Additional information, tables, and figures.

## Abbreviations

ABCD: airway, breathing, circulation, disability
ADT: admission, discharge, and transfer
ATC: Anatomical Therapeutic Chemical
AUPRC: area under the precision-recall curve
AUROC: area under the receiver operating characteristic curve
ECE: expected calibration error
ECI: Elixhauser Comorbidity Index
EDI: Epic Deterioration Index
EHR: electronic health record
FDR: false discovery rate
GCS: Glasgow Coma Score
ICD: International Classification of Diseases
ICU: intensive care unit
ISS: Injury Severity Score
ML: machine learning
PPV: positive predictive value
PR: precision-recall
PTIM: Parkland Trauma Index of Mortality
ROC: receiver operating characteristic
RTS: Revised Trauma Score
SHAP: Shapley additive explanations
TRISS: Trauma and Injury Severity Score
