# Supplementary material for: Dynamic Prediction of Day Mortality in Patients With Trauma Using a Hybrid Neural Network Model: Model Development and Evaluation Study
Source: J Med Internet Res. 2026 Sep 16;28:e86202. doi: 10.2196/86202 (PMC13628034; doi:10.2196/86202)
Supplement: Supplementary file 1 [file jmir_v28i1e86202_app1.docx]

Supplementary Material

Contents

[Appendix S1 – Model architecture	2](#_Toc227759274)

[Appendix S2 – Technical implementations	4](#_Toc227759275)

[Figure S1	5](#_Toc227759276)

[Figure S2	6](#_Toc227759277)

[Figure S3	7](#_Toc227759278)

[Figure S4	8](#_Toc227759279)

[Figure S5	9](#_Toc227759280)

[Figure S6	10](#_Toc227759281)

[Figure S7	11](#_Toc227759282)

[Figure S8	12](#_Toc227759283)

[Figure S9	12](#_Toc227759284)

[Figure S10	13](#_Toc227759285)

[Figure S11	14](#_Toc227759286)

[Table S1	15](#_Toc227759287)

[Table S2	16](#_Toc227759288)

[Table S3	16](#_Toc227759289)

[Table S4	17](#_Toc227759290)

[Table S5	18](#_Toc227759291)

[Table S6	25](#_Toc227759292)

[Table S7	27](#_Toc227759293)

[References	43](#_Toc227759294)

Appendix S1 – Model architecture
The model processes three input modalities: continuous time series, categorical time series, and static tabular features. Continuous time series inputs, comprising clinical measurements such as vital signs and laboratory values, were projected through a 1-dimensional convolution with a kernel size of 1 and 192 output channels, producing per-timestep embeddings across 116 time steps. Categorical time series inputs were represented as multi-hot count vectors at each timestep and embedded via weighted-sum embedding layers, each mapping to 192 dimensions.
The resulting categorical embeddings were combined with the continuous time series embeddings by element-wise addition, forming 116 temporal tokens of dimension 192. Static categorical features were each embedded into 192-dimensional vectors. Static continuous features were projected through a 1-dimensional convolution with a single input channel, 192 output channels, and a kernel size of 1. The static embeddings were concatenated with the temporal tokens along the sequence dimension, yielding a unified input sequence of 126 tokens (116 temporal + 10 static), where the 10 static tokens comprise 6 categorical features (sex, first hospital, and 4 prehospital ABCD assessment categories) and 4 continuous features (age, Elixhauser comorbidity score, height, and weight).
The unified sequence was then processed by 4 transformer encoder layers, each applying multi-head self-attention with 16 heads followed by a position-wise feed-forward network with a hidden size of 768. These feed-forward networks used the Gaussian Error Linear Unit (GELU) as activation function. The model incorporated residual connections, dropout (0.1), and layer normalization. Causal attention masking was applied such that each temporal token attends only to itself, preceding temporal positions, and the static context tokens, preventing information leakage from future observations. Static tokens were constrained to attend only to other static tokens, blocking an information pathway through which future temporal information could propagate to earlier positions via the static representations.

For classification, a shared-weight per-timestep prediction head was applied independently at each temporal position, producing mortality risk logits at every timestep throughout the patient trajectory. This enables continuous risk monitoring without requiring sequence-level aggregation. The prediction head comprised 2 fully connected layers with dimensions 192 to 96 to 1, employed GELU as activation function, and applied dropout (0.55) for regularization. End-of-sequence padding positions were excluded via key padding masks and did not contribute to the loss.

# Appendix S2 – Technical implementations

Using an Nvidia (Santa Clara, California, USA) Tesla V100 GPU (16 GB VRAM) in Microsoft (Redmond, Washington, USA) Azure Machine Learning studio with 6 CPU cores and 112 GB RAM available, the model was implemented using Python 3.10.14, CUDA version 12.1[1], and PyTorch 2.3.1[2]. Scikit-learn 1.7.2[3] was used for evaluation metrics and other machine-learning utilities. SHAP 0.49.1[4] was used for model explainability analysis. We used Optuna 4.7.0[5] for hyper-parameter optimization with configuration management via YAML.

# Figure S1

**Figure S1.** Capital Region prehospital trauma visitation criteria[6]

# Figure S2

**Figure S2.** The dataset creation process with indication of variable sources and dataset sizes.

# Figure S3

**Figure S3.** Receiver operator characteristics curve and precision recall curve for the holdout evaluation summarized by area under the curve (AUC) and area under precision-recall curve (AP). The dashed line in the precision-recall plot indicates the outcome prevalence (3.7%), representing the performance of a non-informative classifier.

# Figure S4

**Figure S4.** Sensitivity for each timestep by predicting mortality for top 5, 10, 15, 20 and 25 percent of probabilities.

# Figure S5

**Figure S5.** Calibration metrics over time comparing raw model outputs with post-hoc calibrated outputs using isotonic regression and Platt scaling. Top panel: Expected Calibration Error (ECE) at selected timepoints (1, 6, 12, 24, 48, 60, and 72 hours). Bottom panel: Brier score at the same timepoints. Dashed grey lines represent raw (uncalibrated) model outputs. Calibration models were fitted per timepoint on the training/validation set and applied to the holdout set.

# Figure S6

**Figure S6.** Reliability diagrams comparing raw model outputs (dashed grey) with isotonic-calibrated outputs (blue) at selected evaluation timepoints (1 hour, 6 hours, 12 hours, 3 days, 7 days, and 14 days). The dashed diagonal represents perfect calibration. Isotonic regression was fitted per timepoint on the training/validation set and applied to the holdout set. Brier scores for raw and calibrated outputs are reported in the 1-hour panel legend. The number of observations per bin varies across timepoints due to decreasing active cohort size.

# Figure S7

**Figure S7.** Predicted mortality probability distributions stratified by outcome (survived, deceased) at selected evaluation timepoints using population-level evaluation (n=1762 survived, n=67 deceased). (A) Distribution over hours (1h, 6h, 12h, 24h, 48h, 72h). (B) Distribution over days (3d, 7d, 14d, 30d). Black horizontal lines indicate the median predicted probability for deceased patients.

# Figure S8

**Figure S8.** Confusion matrices with absolute and normalized values using threshold optimization (threshold=0.040) from internal validation datasets using F5-score.

# Figure S9

**Figure S9.** Confusion matrices with absolute and normalized values using threshold optimization (threshold=0.500) from internal validation datasets using F1-score.

# Figure S10

**Figure S10.** Comparison of discriminative performance between the hybrid neural network (HNN) and TRISS on the subset of holdout patients with complete TRISS inputs (N=878). (A) AUROC and AUPRC over the first 72 hours with 95% confidence intervals. (B) AUROC and AUPRC over 30 days. (C) Number of active patients (left axis) and 30-day mortality prevalence among the active cohort (right axis, %) over the first 72 hours. (D) Active patients and prevalence over 30 days.

# Figure S11

**Figure S11.** Comparison of discriminative performance between the hybrid neural network (HNN) and Revised Trauma Score (RTS) on the subset of holdout patients with complete RTS inputs (N=1401). (A) AUROC and AUPRC over the first 72 hours with 95% confidence intervals. (B) AUROC and AUPRC over 30 days. (C) Number of active patients (left axis) and 30-day mortality prevalence among the active cohort (right axis, %) over the first 72 hours. (D) Active patients and prevalence over 30 days.

# Table S1

| Group name | ATC-codes |
| --- | --- |
| Cardiovascular drugs | C01, C02, C07 |
| Antibiotics | J01 |
| Neuro drugs | N05, M03 |
| Anti-thrombotics | B01 |
| Dieurotics | C03 |
| Hemostatics | B02 |
| Infusion | B05B, B05X |
| Blood | B05A |
| Opiods | N02A |
| Local anastethics | N01B |
| Anastethics | N01A |
| Insulin | A10A |

**Table S1.** Medication group names and their corresponding Anatomical Therapeutic Chemical (ATC) Classification System codes. All sub-codes to the presented codes were included in the corresponding group.

# Table S2

| Group name | EHR Variable name |
| --- | --- |
| Lactate | ’LAKTAT(POC);P(AB)','LAKTAT;P(AB)','LAKTAT;P(VB)','LAKTAT(POC);P(VB)', 'LAKTAT;CSV','LAKTAT(POC);CSV','LAKTAT(POC);P(KB)' |
| Base Excess | 'BASE EXCESS;ECV', 'ECV-BASE EXCESS;(POC)' |
| Hemoglobin | 'HÆMOGLOBIN;B', 'HÆMOGLOBIN(POC);B', 'HÆMOGLOBIN (POC);B' |
| Leukocytes | 'LEUKOCYTTER;B' |
| TEG-Reaction Time | 'TEG-R' |
| TEG-Maximum Amplitude | ’TEG-MA' |
| TEG-Fibronolysis | ’TEG-LY30' |

**Table S2.** Laboratory result group and their electronic health record (EHR) variable names.

# Table S3

| Timestamp from trajectory start | Binning frequency |
| --- | --- |
| 6 hours | 10 minutes |
| 6 to 12 hours | 20 minutes |
| 12 to 24 hours | 1 hour |
| 24 to 72 hours | 4 hours |
| 72 hours to 14 days | 12 hours |
| 14 days to 30 days | 24 hours |

**Table S3.** An overview of the temporally determined data segments and their corresponding binning frequency.

# Table S4

| A: Airway | B: Breathing | C: Circulation | D: Disability |
| --- | --- | --- | --- |
| Clear | Normal | Normal | Awake |
| Threatened | Lightly Affected | Lightly Affected | Reduced Awareness |
| Blocked | Very Affected | Very Affected | Unconscious |
|  | Respiratory Arrest | Cardiac Arrest |  |

**Table S4.** The possible values for the prehospital Airway, Breathing, Circulation, Disability classification

# Table S5

|  | | **Grouped by Dataset** | | | | |
| --- | --- | --- | --- | --- | --- | --- |
|  |  | **Missing** | **Overall** | **Development (training)** | **Holdout evaluation** | **P-Value** |
| **n** |  |  | 9496 | 7667 | 1829 |  |
| **Age, years, mean (SD)** |  | 0 | 42.2 (22.2) | 41.8 (22.2) | 43.6 (22.5) | 0.002† |
| **Sex, n (%)** | **Female** | 0 | 3188 (33.6) | 2548 (33.2) | 640 (35.0) | 0.161‡ |
|  | **Male** |  | 6308 (66.4) | 5119 (66.8) | 1189 (65.0) |  |
| **Trajectory duration, days, mean (SD)** |  | 0 | 7.6 (21.5) | 8.3 (23.0) | 5.0 (13.8) | <0.001† |
| **Elixhauser Comorbidity Index, mean (SD)** |  | 0 | 0.7 (3.2) | 0.7 (3.1) | 0.8 (3.5) | 0.203† |
| **Height, cm, mean (SD)** |  | 3895 | 168.8 (25.0) | 169.1 (24.8) | 167.8 (25.6) | 0.132† |
| **Weight, kg, mean (SD)** |  | 3223 | 72.2 (23.7) | 72.2 (24.0) | 72.1 (22.8) | 0.841† |
| **Injury Severity Score, mean (SD)** |  | 3941 | 6.6 (10.6) | 6.6 (10.8) | 6.5 (9.9) | 0.573† |
| **Glasgow Coma Score, median [Q1,Q3]** |  | 116 | 15.0 [13.0,15.0] | 15.0 [12.0,15.0] | 15.0 [14.0,15.0] | <0.001§ |
| **EWS score, mean (SD)** |  | 3840 | 3.1 (2.8) | 3.2 (2.8) | 2.7 (2.8) | <0.001† |
| **Heart rate (agg. mean), bpm, mean (SD)** |  | 31 | 85.9 (16.3) | 85.9 (16.2) | 86.2 (16.7) | 0.512† |
| **Respiratory rate (agg. mean), /min, median [Q1,Q3]** |  | 1708 | 16.4 [15.4,18.0] | 16.3 [15.3,18.0] | 16.6 [15.7,18.0] | <0.001§ |
| **Systolic blood pressure (agg. mean), mmHg, mean (SD)** |  | 47 | 126.1 (19.4) | 125.8 (19.4) | 127.6 (19.3) | <0.001† |
| **Oxygen saturation (agg. mean), %, median [Q1,Q3]** |  | 32 | 97.5 [96.1,98.5] | 97.5 [96.2,98.5] | 97.5 [96.0,98.6] | 0.678§ |
| **Temperature (agg. mean), °C, median [Q1,Q3]** |  | 2495 | 36.9 [36.6,37.2] | 36.9 [36.6,37.2] | 36.8 [36.5,37.1] | <0.001§ |
| **Anesthetics (count of prescriptions), mean [min,max]** |  | 0 | 3.9 [0.0,199.0] | 4.2 [0.0,155.0] | 2.6 [0.0,199.0] | <0.001† |
| **Antibiotics (count of prescriptions), mean [min,max]** |  | 0 | 12.8 [0.0,882.0] | 14.0 [0.0,882.0] | 7.7 [0.0,289.0] | <0.001† |
| **Anti-thrombotic agents (count of prescriptions), mean [min,max]** |  | 0 | 5.5 [0.0,601.0] | 6.0 [0.0,601.0] | 3.4 [0.0,228.0] | <0.001† |
| **Blood products (count of prescriptions), mean [min,max]** |  | 0 | 0.2 [0.0,27.0] | 0.2 [0.0,27.0] | 0.1 [0.0,15.0] | <0.001† |
| **Cardiovascular drugs (count of prescriptions), mean [min,max]** |  | 0 | 5.2 [0.0,839.0] | 5.7 [0.0,839.0] | 3.2 [0.0,405.0] | <0.001† |
| **Diuretics (count of prescriptions), mean [min,max]** |  | 0 | 2.2 [0.0,496.0] | 2.5 [0.0,496.0] | 1.4 [0.0,170.0] | <0.001† |
| **Hemostatics (count of prescriptions), mean [min,max]** |  | 0 | 0.4 [0.0,94.0] | 0.4 [0.0,94.0] | 0.3 [0.0,16.0] | <0.001† |
| **Infusion (count of prescriptions), mean [min,max]** |  | 0 | 4.4 [0.0,584.0] | 4.8 [0.0,584.0] | 2.6 [0.0,354.0] | <0.001† |
| **Insulin (count of prescriptions), mean [min,max]** |  | 0 | 1.5 [0.0,627.0] | 1.5 [0.0,627.0] | 1.6 [0.0,441.0] | 0.843† |
| **Local anesthetics (count of prescriptions), mean [min,max]** |  | 0 | 0.6 [0.0,57.0] | 0.7 [0.0,57.0] | 0.5 [0.0,20.0] | <0.001† |
| **Neurological drugs (count of prescriptions), mean [min,max]** |  | 0 | 10.9 [0.0,903.0] | 12.1 [0.0,903.0] | 6.1 [0.0,728.0] | <0.001† |
| **Opioids (count of prescriptions), mean [min,max]** |  | 0 | 13.0 [0.0,617.0] | 14.0 [0.0,617.0] | 8.7 [0.0,285.0] | <0.001† |
| **Base Excess, mmol/L, mean (SD)** |  | 4074 | 1.3 (4.0) | 1.5 (4.1) | 0.8 (3.4) | <0.001† |
| **Lactate, mmol/L, median [Q1,Q3]** |  | 2269 | 2.2 [1.6,3.2] | 2.2 [1.6,3.3] | 2.1 [1.5,3.1] | 0.032§ |
| **Hemoglobin, mmol/L, mean (SD)** |  | 711 | 8.7 (1.0) | 8.7 (1.0) | 8.7 (1.0) | 0.464† |
| **Leukocytes count, 10⁹/L, median [Q1,Q3]** |  | 811 | 10.3 [7.7,14.2] | 10.5 [7.8,14.4] | 9.5 [7.3,13.1] | <0.001§ |
| **TEG Reaction time, min, median [Q1,Q3]** |  | 8279 | 5.3 [4.7,6.3] | 5.4 [4.7,6.4] | 5.2 [4.3,6.0] | <0.001§ |
| **TEG Maximum amplitude, mm, mean (SD)** |  | 8279 | 65.4 (6.9) | 64.8 (6.9) | 67.3 (6.7) | <0.001† |
| **TEG Fibrinolysis (LY30), %, median [Q1,Q3]** |  | 8393 | 0.3 [0.0,1.0] | 0.2 [0.0,1.0] | 0.3 [0.0,0.9] | 0.734§ |
| **A: Airway, n (%)** | **Clear** | 1165 | 7970 (95.7) | 6414 (95.3) | 1556 (97.2) | 0.003‡ |
|  | **Threatened** |  | 327 (3.9) | 285 (4.2) | 42 (2.6) |  |
|  | **Blocked** |  | 34 (0.4) | 31 (0.5) | 3 (0.2) |  |
| **B: Breathing, n (%)** | **Normal** | 1151 | 7012 (84.0) | 5622 (83.4) | 1390 (86.8) | 0.001‡ |
|  | **Lightly affected** |  | 1029 (12.3) | 854 (12.7) | 175 (10.9) |  |
|  | **Very affected** |  | 236 (2.8) | 209 (3.1) | 27 (1.7) |  |
|  | **Respiratory arrest (ceased)** |  | 68 (0.8) | 59 (0.9) | 9 (0.6) |  |
| **C: Circulation, n (%)** | **Normal** | 1158 | 6996 (83.9) | 5624 (83.5) | 1372 (85.6) | 0.043‡ |
|  | **Lightly affected** |  | 1018 (12.2) | 832 (12.4) | 186 (11.6) |  |
|  | **Very affected** |  | 255 (3.1) | 219 (3.3) | 36 (2.2) |  |
|  | **Cardiac arrest** |  | 69 (0.8) | 61 (0.9) | 8 (0.5) |  |
| **D: Disability, n (%)** | **Awake** | 1172 | 6703 (80.5) | 5337 (79.3) | 1366 (85.5) | <0.001‡ |
|  | **Reduced awareness** |  | 1237 (14.9) | 1047 (15.6) | 190 (11.9) |  |
|  | **Unconscious** |  | 384 (4.6) | 342 (5.1) | 42 (2.6) |  |
| **Level 1  Trauma Bay visit, n (%)** | **No** | 0 | 4866 (51.2) | 3648 (47.6) | 1218 (66.6) | <0.001‡ |
|  | **Yes** |  | 4630 (48.8) | 4019 (52.4) | 611 (33.4) |  |
| **Operating room visit, n (%)** | **No** | 0 | 7228 (76.1) | 5702 (74.4) | 1526 (83.4) | <0.001‡ |
|  | **Yes** |  | 2268 (23.9) | 1965 (25.6) | 303 (16.6) |  |
| **ICU admission, n (%)** | **No** | 0 | 7902 (83.2) | 6338 (82.7) | 1564 (85.5) | 0.004‡ |
|  | **Yes** |  | 1594 (16.8) | 1329 (17.3) | 265 (14.5) |  |
| **Ward admission, n (%)** | **No** | 0 | 4337 (45.7) | 3299 (43.0) | 1038 (56.8) | <0.001‡ |
|  | **Yes** |  | 5159 (54.3) | 4368 (57.0) | 791 (43.2) |  |
| **Deceased 30-day, n (%)** | **Deceased** | 0 | 372 (3.9) | 305 (4.0) | 67 (3.7) | 0.578‡ |
|  | **Survived** |  | 9124 (96.1) | 7362 (96.0) | 1762 (96.3) |  |

**Table S5.** Patient characteristics stratified on dataset split. *P*-value tests: † Welch’s T-test; ‡ Chi-squared; § Kruskal-Wallis. TEG: Thrombelastography, EWS: Early Warning Score

# Table S6

| **Time** | **AUROC (Full)**  **[CI]** | **AUPRC (Full)**  **[CI]** | **N (Full)** | **AUROC (Active)**  **[CI]** | **AUPRC (Active)**  **[CI]** | **N (Active)** |
| --- | --- | --- | --- | --- | --- | --- |
| 1 h | 0.905 [0.856–0.953] | 0.388 [0.284–0.518] | 1829 | 0.905 [0.856–0.953] | 0.388 [0.284–0.518] | 1829 |
| 3 h | 0.928 [0.885–0.971] | 0.434 [0.325–0.568] | 1829 | 0.926 [0.881–0.970] | 0.428 [0.320–0.564] | 1666 |
| 6 h | 0.936 [0.895–0.977] | 0.454 [0.341–0.583] | 1829 | 0.915 [0.868–0.963] | 0.452 [0.336–0.585] | 1176 |
| 12 h | 0.936 [0.896–0.977] | 0.461 [0.346–0.598] | 1829 | 0.887 [0.829–0.945] | 0.375 [0.260–0.534] | 914 |
| 24 h | 0.937 [0.897–0.978] | 0.519 [0.407–0.649] | 1829 | 0.874 [0.808–0.940] | 0.409 [0.278–0.555] | 729 |
| 48 h | 0.949 [0.913–0.986] | 0.560 [0.451–0.678] | 1829 | 0.896 [0.826–0.966] | 0.407 [0.261–0.569] | 584 |
| 72 h | 0.954 [0.918–0.989] | 0.557 [0.443–0.680] | 1829 | 0.919 [0.851–0.987] | 0.409 [0.267–0.580] | 506 |
| 7 days | 0.957 [0.923–0.991] | 0.601 [0.490–0.709] | 1829 | 0.921 [0.833–1.000] | 0.316 [0.183–0.570] | 324 |
| 14 days | 0.960 [0.927–0.993] | 0.633 [0.524–0.743] | 1829 | 0.905 [0.778–1.000] | 0.350 [0.147–0.682] | 162 |
| 30 days | 0.962 [0.930–0.994] | 0.655 [0.545–0.765] | 1829 | NA | NA | 52 |

**Table S6.** AUROC and AUPRC with 95% confidence intervals at selected evaluation timepoints for population-level (full cohort, N=1829) and active-cohort evaluation strategies. Confidence intervals for AUROC were calculated using the DeLong method; confidence intervals for AUPRC were calculated by bootstrapping. N (Active) denotes the number of patients with trajectories extending beyond each timepoint. Active-cohort metrics at 30 days are not reported due to insufficient sample size (n=52, with fewer than 2 outcome events).

# Table S7

| **Timeframe** | **Feature** | **Mean absolute SHAP** | **Standard deviation absolute SHAP** | **Number of patients** |
| --- | --- | --- | --- | --- |
| 1H | BASE_EXCESS_max | 0.00177 | 0.02954 | 500 |
| 1H | EWS_SCORE_max | 0.00021 | 0.00405 | 500 |
| 1H | GCS_min | 0.04839 | 0.14111 | 500 |
| 1H | HEMOGLOBIN_max | 0.00250 | 0.02680 | 500 |
| 1H | HR_mean | 0.00832 | 0.01977 | 500 |
| 1H | HR_std | 0.01380 | 0.05204 | 500 |
| 1H | ISS_computed_max | 0.00003 | 0.00058 | 500 |
| 1H | ISS_notes_max | 0.00387 | 0.05791 | 500 |
| 1H | LACTATE_max | 0.00405 | 0.08227 | 500 |
| 1H | LEUKOCYTES_max | 0.00358 | 0.04338 | 500 |
| 1H | RESPIRATORYRATE_mean | 0.00065 | 0.01006 | 500 |
| 1H | RESPIRATORYRATE_std | 0.00008 | 0.00173 | 500 |
| 1H | SBP_mean | 0.00508 | 0.01457 | 500 |
| 1H | SBP_std | 0.00792 | 0.02454 | 500 |
| 1H | SPO2_mean | 0.02404 | 0.11667 | 500 |
| 1H | SPO2_std | 0.00932 | 0.04625 | 500 |
| 1H | TEG-LY30_max | 0.00007 | 0.00106 | 500 |
| 1H | TEG-MA_max | 0.00009 | 0.00168 | 500 |
| 1H | TEG-R_max | 0.00013 | 0.00184 | 500 |
| 1H | TEMP_mean | 0.00057 | 0.01008 | 500 |
| 1H | TEMP_std | 0.00006 | 0.00129 | 500 |
| 1H | static_cat:SEX | 0.04127 |  | 500 |
| 1H | static_cat:FIRST_HOSPITAL | 0.08770 |  | 500 |
| 1H | static_cat:A | 0.03603 |  | 500 |
| 1H | static_cat:B | 0.10409 |  | 500 |
| 1H | static_cat:C | 0.05470 |  | 500 |
| 1H | static_cat:D | 0.13610 |  | 500 |
| 1H | static_cat:HEIGHT_na | 0.04410 |  | 500 |
| 1H | static_cat:WEIGHT_na | 0.07982 |  | 500 |
| 1H | static_cont:AGE | 0.17129 |  | 500 |
| 1H | static_cont:ASMT_ELIX | 0.12017 |  | 500 |
| 1H | static_cont:HEIGHT | 0.08621 |  | 500 |
| 1H | static_cont:WEIGHT | 0.10401 |  | 500 |
| 1H | cat_ts:medication:anastethics | 0.00174 |  | 500 |
| 1H | cat_ts:medication:anti_thrombotic | 0.00001 |  | 500 |
| 1H | cat_ts:medication:antibiotics | 0.00028 |  | 500 |
| 1H | cat_ts:medication:blood | 0.00000 |  | 500 |
| 1H | cat_ts:medication:cardiovascular_drugs | 0.00000 |  | 500 |
| 1H | cat_ts:medication:diuretics | 0.00000 |  | 500 |
| 1H | cat_ts:medication:hemostatics | 0.00068 |  | 500 |
| 1H | cat_ts:medication:infusion | 0.00126 |  | 500 |
| 1H | cat_ts:medication:insulin | 0.00000 |  | 500 |
| 1H | cat_ts:medication:local_anastethics | 0.00000 |  | 500 |
| 1H | cat_ts:medication:neuro_drugs | 0.00117 |  | 500 |
| 1H | cat_ts:medication:opiods | 0.00004 |  | 500 |
| 1H | cat_ts:ADT:BED | 0.00000 |  | 500 |
| 1H | cat_ts:ADT:ICU | 0.00005 |  | 500 |
| 1H | cat_ts:ADT:OR | 0.00000 |  | 500 |
| 1H | cat_ts:ADT:TC | 0.00280 |  | 500 |
| 6H | BASE_EXCESS_max | 0.00805 | 0.02258 | 314 |
| 6H | EWS_SCORE_max | 0.00244 | 0.01060 | 314 |
| 6H | GCS_min | 0.03402 | 0.04693 | 314 |
| 6H | HEMOGLOBIN_max | 0.00500 | 0.01285 | 314 |
| 6H | HR_mean | 0.00971 | 0.00954 | 314 |
| 6H | HR_std | 0.01379 | 0.01846 | 314 |
| 6H | ISS_computed_max | 0.00097 | 0.00798 | 314 |
| 6H | ISS_notes_max | 0.00892 | 0.04794 | 314 |
| 6H | LACTATE_max | 0.02250 | 0.06914 | 314 |
| 6H | LEUKOCYTES_max | 0.01566 | 0.03305 | 314 |
| 6H | RESPIRATORYRATE_mean | 0.00394 | 0.01753 | 314 |
| 6H | RESPIRATORYRATE_std | 0.00076 | 0.00661 | 314 |
| 6H | SBP_mean | 0.01130 | 0.01390 | 314 |
| 6H | SBP_std | 0.00646 | 0.00914 | 314 |
| 6H | SPO2_mean | 0.02429 | 0.05277 | 314 |
| 6H | SPO2_std | 0.01181 | 0.02904 | 314 |
| 6H | TEG-LY30_max | 0.00599 | 0.08911 | 314 |
| 6H | TEG-MA_max | 0.00065 | 0.00244 | 314 |
| 6H | TEG-R_max | 0.00055 | 0.00182 | 314 |
| 6H | TEMP_mean | 0.00812 | 0.01829 | 314 |
| 6H | TEMP_std | 0.00018 | 0.00096 | 314 |
| 6H | static_cat:SEX | 0.08602 |  | 314 |
| 6H | static_cat:FIRST_HOSPITAL | 0.12580 |  | 314 |
| 6H | static_cat:A | 0.08293 |  | 314 |
| 6H | static_cat:B | 0.16480 |  | 314 |
| 6H | static_cat:C | 0.12627 |  | 314 |
| 6H | static_cat:D | 0.20045 |  | 314 |
| 6H | static_cat:HEIGHT_na | 0.09768 |  | 314 |
| 6H | static_cat:WEIGHT_na | 0.08294 |  | 314 |
| 6H | static_cont:AGE | 0.34353 |  | 314 |
| 6H | static_cont:ASMT_ELIX | 0.25367 |  | 314 |
| 6H | static_cont:HEIGHT | 0.20101 |  | 314 |
| 6H | static_cont:WEIGHT | 0.19980 |  | 314 |
| 6H | cat_ts:medication:anastethics | 0.01561 |  | 314 |
| 6H | cat_ts:medication:anti_thrombotic | 0.00105 |  | 314 |
| 6H | cat_ts:medication:antibiotics | 0.00808 |  | 314 |
| 6H | cat_ts:medication:blood | 0.00004 |  | 314 |
| 6H | cat_ts:medication:cardiovascular_drugs | 0.02670 |  | 314 |
| 6H | cat_ts:medication:diuretics | 0.00077 |  | 314 |
| 6H | cat_ts:medication:hemostatics | 0.01201 |  | 314 |
| 6H | cat_ts:medication:infusion | 0.03574 |  | 314 |
| 6H | cat_ts:medication:insulin | 0.00013 |  | 314 |
| 6H | cat_ts:medication:local_anastethics | 0.00161 |  | 314 |
| 6H | cat_ts:medication:neuro_drugs | 0.00757 |  | 314 |
| 6H | cat_ts:medication:opiods | 0.00950 |  | 314 |
| 6H | cat_ts:ADT:BED | 0.00355 |  | 314 |
| 6H | cat_ts:ADT:ICU | 0.00768 |  | 314 |
| 6H | cat_ts:ADT:OR | 0.00246 |  | 314 |
| 6H | cat_ts:ADT:TC | 0.00675 |  | 314 |
| 12H | BASE_EXCESS_max | 0.00877 | 0.02195 | 237 |
| 12H | EWS_SCORE_max | 0.00391 | 0.01638 | 237 |
| 12H | GCS_min | 0.02898 | 0.03342 | 237 |
| 12H | HEMOGLOBIN_max | 0.00492 | 0.01121 | 237 |
| 12H | HR_mean | 0.00846 | 0.00749 | 237 |
| 12H | HR_std | 0.01238 | 0.01497 | 237 |
| 12H | ISS_computed_max | 0.00160 | 0.01886 | 237 |
| 12H | ISS_notes_max | 0.01649 | 0.08607 | 237 |
| 12H | LACTATE_max | 0.02223 | 0.06350 | 237 |
| 12H | LEUKOCYTES_max | 0.01838 | 0.03855 | 237 |
| 12H | RESPIRATORYRATE_mean | 0.00239 | 0.00605 | 237 |
| 12H | RESPIRATORYRATE_std | 0.00059 | 0.00166 | 237 |
| 12H | SBP_mean | 0.01207 | 0.01456 | 237 |
| 12H | SBP_std | 0.00622 | 0.00795 | 237 |
| 12H | SPO2_mean | 0.02079 | 0.02941 | 237 |
| 12H | SPO2_std | 0.00940 | 0.01399 | 237 |
| 12H | TEG-LY30_max | 0.00606 | 0.07001 | 237 |
| 12H | TEG-MA_max | 0.00088 | 0.00364 | 237 |
| 12H | TEG-R_max | 0.00087 | 0.00291 | 237 |
| 12H | TEMP_mean | 0.00868 | 0.01239 | 237 |
| 12H | TEMP_std | 0.00093 | 0.00749 | 237 |
| 12H | static_cat:SEX | 0.09250 |  | 237 |
| 12H | static_cat:FIRST_HOSPITAL | 0.12646 |  | 237 |
| 12H | static_cat:A | 0.08288 |  | 237 |
| 12H | static_cat:B | 0.15556 |  | 237 |
| 12H | static_cat:C | 0.14014 |  | 237 |
| 12H | static_cat:D | 0.27192 |  | 237 |
| 12H | static_cat:HEIGHT_na | 0.11613 |  | 237 |
| 12H | static_cat:WEIGHT_na | 0.09928 |  | 237 |
| 12H | static_cont:AGE | 0.37667 |  | 237 |
| 12H | static_cont:ASMT_ELIX | 0.32446 |  | 237 |
| 12H | static_cont:HEIGHT | 0.22687 |  | 237 |
| 12H | static_cont:WEIGHT | 0.22332 |  | 237 |
| 12H | cat_ts:medication:anastethics | 0.02418 |  | 237 |
| 12H | cat_ts:medication:anti_thrombotic | 0.00047 |  | 237 |
| 12H | cat_ts:medication:antibiotics | 0.01674 |  | 237 |
| 12H | cat_ts:medication:blood | 0.00021 |  | 237 |
| 12H | cat_ts:medication:cardiovascular_drugs | 0.03884 |  | 237 |
| 12H | cat_ts:medication:diuretics | 0.00181 |  | 237 |
| 12H | cat_ts:medication:hemostatics | 0.02631 |  | 237 |
| 12H | cat_ts:medication:infusion | 0.05353 |  | 237 |
| 12H | cat_ts:medication:insulin | 0.00115 |  | 237 |
| 12H | cat_ts:medication:local_anastethics | 0.00350 |  | 237 |
| 12H | cat_ts:medication:neuro_drugs | 0.00879 |  | 237 |
| 12H | cat_ts:medication:opiods | 0.05638 |  | 237 |
| 12H | cat_ts:ADT:BED | 0.00926 |  | 237 |
| 12H | cat_ts:ADT:ICU | 0.00895 |  | 237 |
| 12H | cat_ts:ADT:OR | 0.00298 |  | 237 |
| 12H | cat_ts:ADT:TC | 0.00850 |  | 237 |
| 1D | BASE_EXCESS_max | 0.00705 | 0.01465 | 191 |
| 1D | EWS_SCORE_max | 0.00393 | 0.00998 | 191 |
| 1D | GCS_min | 0.02823 | 0.02843 | 191 |
| 1D | HEMOGLOBIN_max | 0.00421 | 0.00629 | 191 |
| 1D | HR_mean | 0.00823 | 0.00715 | 191 |
| 1D | HR_std | 0.01207 | 0.01163 | 191 |
| 1D | ISS_computed_max | 0.00055 | 0.00190 | 191 |
| 1D | ISS_notes_max | 0.01683 | 0.07784 | 191 |
| 1D | LACTATE_max | 0.01730 | 0.04768 | 191 |
| 1D | LEUKOCYTES_max | 0.01603 | 0.02350 | 191 |
| 1D | RESPIRATORYRATE_mean | 0.00356 | 0.00846 | 191 |
| 1D | RESPIRATORYRATE_std | 0.00114 | 0.00368 | 191 |
| 1D | SBP_mean | 0.01152 | 0.01136 | 191 |
| 1D | SBP_std | 0.00579 | 0.00603 | 191 |
| 1D | SPO2_mean | 0.01796 | 0.02746 | 191 |
| 1D | SPO2_std | 0.00854 | 0.01210 | 191 |
| 1D | TEG-LY30_max | 0.01039 | 0.12087 | 191 |
| 1D | TEG-MA_max | 0.00118 | 0.00371 | 191 |
| 1D | TEG-R_max | 0.00090 | 0.00307 | 191 |
| 1D | TEMP_mean | 0.00861 | 0.00966 | 191 |
| 1D | TEMP_std | 0.00178 | 0.01349 | 191 |
| 1D | static_cat:SEX | 0.09222 |  | 191 |
| 1D | static_cat:FIRST_HOSPITAL | 0.12736 |  | 191 |
| 1D | static_cat:A | 0.09508 |  | 191 |
| 1D | static_cat:B | 0.16409 |  | 191 |
| 1D | static_cat:C | 0.16042 |  | 191 |
| 1D | static_cat:D | 0.25434 |  | 191 |
| 1D | static_cat:HEIGHT_na | 0.11957 |  | 191 |
| 1D | static_cat:WEIGHT_na | 0.08860 |  | 191 |
| 1D | static_cont:AGE | 0.36679 |  | 191 |
| 1D | static_cont:ASMT_ELIX | 0.34133 |  | 191 |
| 1D | static_cont:HEIGHT | 0.28625 |  | 191 |
| 1D | static_cont:WEIGHT | 0.23644 |  | 191 |
| 1D | cat_ts:medication:anastethics | 0.02407 |  | 191 |
| 1D | cat_ts:medication:anti_thrombotic | 0.00507 |  | 191 |
| 1D | cat_ts:medication:antibiotics | 0.02143 |  | 191 |
| 1D | cat_ts:medication:blood | 0.00034 |  | 191 |
| 1D | cat_ts:medication:cardiovascular_drugs | 0.04176 |  | 191 |
| 1D | cat_ts:medication:diuretics | 0.02335 |  | 191 |
| 1D | cat_ts:medication:hemostatics | 0.03393 |  | 191 |
| 1D | cat_ts:medication:infusion | 0.04579 |  | 191 |
| 1D | cat_ts:medication:insulin | 0.00526 |  | 191 |
| 1D | cat_ts:medication:local_anastethics | 0.00479 |  | 191 |
| 1D | cat_ts:medication:neuro_drugs | 0.00897 |  | 191 |
| 1D | cat_ts:medication:opiods | 0.05217 |  | 191 |
| 1D | cat_ts:ADT:BED | 0.01229 |  | 191 |
| 1D | cat_ts:ADT:ICU | 0.00923 |  | 191 |
| 1D | cat_ts:ADT:OR | 0.00550 |  | 191 |
| 1D | cat_ts:ADT:TC | 0.00898 |  | 191 |
| 3D | BASE_EXCESS_max | 0.00650 | 0.01415 | 127 |
| 3D | EWS_SCORE_max | 0.00686 | 0.01443 | 127 |
| 3D | GCS_min | 0.02436 | 0.01762 | 127 |
| 3D | HEMOGLOBIN_max | 0.00440 | 0.01303 | 127 |
| 3D | HR_mean | 0.00720 | 0.00473 | 127 |
| 3D | HR_std | 0.01125 | 0.01035 | 127 |
| 3D | ISS_computed_max | 0.00049 | 0.00130 | 127 |
| 3D | ISS_notes_max | 0.01672 | 0.06896 | 127 |
| 3D | LACTATE_max | 0.01104 | 0.02579 | 127 |
| 3D | LEUKOCYTES_max | 0.01267 | 0.01475 | 127 |
| 3D | RESPIRATORYRATE_mean | 0.00420 | 0.00669 | 127 |
| 3D | RESPIRATORYRATE_std | 0.00128 | 0.00169 | 127 |
| 3D | SBP_mean | 0.01045 | 0.00993 | 127 |
| 3D | SBP_std | 0.00600 | 0.00820 | 127 |
| 3D | SPO2_mean | 0.01490 | 0.01902 | 127 |
| 3D | SPO2_std | 0.00813 | 0.01169 | 127 |
| 3D | TEG-LY30_max | 0.00967 | 0.09238 | 127 |
| 3D | TEG-MA_max | 0.00115 | 0.00266 | 127 |
| 3D | TEG-R_max | 0.00061 | 0.00144 | 127 |
| 3D | TEMP_mean | 0.00747 | 0.00634 | 127 |
| 3D | TEMP_std | 0.00167 | 0.00467 | 127 |
| 3D | static_cat:SEX | 0.10413 |  | 127 |
| 3D | static_cat:FIRST_HOSPITAL | 0.13479 |  | 127 |
| 3D | static_cat:A | 0.09549 |  | 127 |
| 3D | static_cat:B | 0.15413 |  | 127 |
| 3D | static_cat:C | 0.13246 |  | 127 |
| 3D | static_cat:D | 0.29584 |  | 127 |
| 3D | static_cat:HEIGHT_na | 0.08478 |  | 127 |
| 3D | static_cat:WEIGHT_na | 0.05660 |  | 127 |
| 3D | static_cont:AGE | 0.43605 |  | 127 |
| 3D | static_cont:ASMT_ELIX | 0.44607 |  | 127 |
| 3D | static_cont:HEIGHT | 0.28878 |  | 127 |
| 3D | static_cont:WEIGHT | 0.25658 |  | 127 |
| 3D | cat_ts:medication:anastethics | 0.02787 |  | 127 |
| 3D | cat_ts:medication:anti_thrombotic | 0.01102 |  | 127 |
| 3D | cat_ts:medication:antibiotics | 0.02349 |  | 127 |
| 3D | cat_ts:medication:blood | 0.00336 |  | 127 |
| 3D | cat_ts:medication:cardiovascular_drugs | 0.04634 |  | 127 |
| 3D | cat_ts:medication:diuretics | 0.02582 |  | 127 |
| 3D | cat_ts:medication:hemostatics | 0.03052 |  | 127 |
| 3D | cat_ts:medication:infusion | 0.03775 |  | 127 |
| 3D | cat_ts:medication:insulin | 0.00775 |  | 127 |
| 3D | cat_ts:medication:local_anastethics | 0.00495 |  | 127 |
| 3D | cat_ts:medication:neuro_drugs | 0.01185 |  | 127 |
| 3D | cat_ts:medication:opiods | 0.03077 |  | 127 |
| 3D | cat_ts:ADT:BED | 0.00878 |  | 127 |
| 3D | cat_ts:ADT:ICU | 0.00880 |  | 127 |
| 3D | cat_ts:ADT:OR | 0.00554 |  | 127 |
| 3D | cat_ts:ADT:TC | 0.00839 |  | 127 |
| 7D | BASE_EXCESS_max | 0.00635 | 0.01043 | 83 |
| 7D | EWS_SCORE_max | 0.00682 | 0.00706 | 83 |
| 7D | GCS_min | 0.02412 | 0.01508 | 83 |
| 7D | HEMOGLOBIN_max | 0.00293 | 0.00376 | 83 |
| 7D | HR_mean | 0.00630 | 0.00387 | 83 |
| 7D | HR_std | 0.01090 | 0.00909 | 83 |
| 7D | ISS_computed_max | 0.00111 | 0.00292 | 83 |
| 7D | ISS_notes_max | 0.02011 | 0.06373 | 83 |
| 7D | LACTATE_max | 0.01300 | 0.03238 | 83 |
| 7D | LEUKOCYTES_max | 0.01238 | 0.01190 | 83 |
| 7D | RESPIRATORYRATE_mean | 0.00442 | 0.00735 | 83 |
| 7D | RESPIRATORYRATE_std | 0.00283 | 0.00343 | 83 |
| 7D | SBP_mean | 0.00948 | 0.00868 | 83 |
| 7D | SBP_std | 0.00508 | 0.00549 | 83 |
| 7D | SPO2_mean | 0.01242 | 0.01125 | 83 |
| 7D | SPO2_std | 0.00728 | 0.00875 | 83 |
| 7D | TEG-LY30_max | 0.01170 | 0.10013 | 83 |
| 7D | TEG-MA_max | 0.00290 | 0.00796 | 83 |
| 7D | TEG-R_max | 0.00090 | 0.00222 | 83 |
| 7D | TEMP_mean | 0.00854 | 0.01066 | 83 |
| 7D | TEMP_std | 0.00556 | 0.01045 | 83 |
| 7D | static_cat:SEX | 0.11955 |  | 83 |
| 7D | static_cat:FIRST_HOSPITAL | 0.15637 |  | 83 |
| 7D | static_cat:A | 0.10408 |  | 83 |
| 7D | static_cat:B | 0.17066 |  | 83 |
| 7D | static_cat:C | 0.14368 |  | 83 |
| 7D | static_cat:D | 0.44464 |  | 83 |
| 7D | static_cat:HEIGHT_na | 0.08537 |  | 83 |
| 7D | static_cat:WEIGHT_na | 0.01976 |  | 83 |
| 7D | static_cont:AGE | 0.45474 |  | 83 |
| 7D | static_cont:ASMT_ELIX | 0.52960 |  | 83 |
| 7D | static_cont:HEIGHT | 0.30985 |  | 83 |
| 7D | static_cont:WEIGHT | 0.29393 |  | 83 |
| 7D | cat_ts:medication:anastethics | 0.03288 |  | 83 |
| 7D | cat_ts:medication:anti_thrombotic | 0.01747 |  | 83 |
| 7D | cat_ts:medication:antibiotics | 0.01723 |  | 83 |
| 7D | cat_ts:medication:blood | 0.00148 |  | 83 |
| 7D | cat_ts:medication:cardiovascular_drugs | 0.04130 |  | 83 |
| 7D | cat_ts:medication:diuretics | 0.03823 |  | 83 |
| 7D | cat_ts:medication:hemostatics | 0.03975 |  | 83 |
| 7D | cat_ts:medication:infusion | 0.03135 |  | 83 |
| 7D | cat_ts:medication:insulin | 0.00634 |  | 83 |
| 7D | cat_ts:medication:local_anastethics | 0.00795 |  | 83 |
| 7D | cat_ts:medication:neuro_drugs | 0.01234 |  | 83 |
| 7D | cat_ts:medication:opiods | 0.02187 |  | 83 |
| 7D | cat_ts:ADT:BED | 0.00497 |  | 83 |
| 7D | cat_ts:ADT:ICU | 0.01042 |  | 83 |
| 7D | cat_ts:ADT:OR | 0.00822 |  | 83 |
| 7D | cat_ts:ADT:TC | 0.00990 |  | 83 |
| 14D | BASE_EXCESS_max | 0.00410 | 0.00437 | 42 |
| 14D | EWS_SCORE_max | 0.00396 | 0.00473 | 42 |
| 14D | GCS_min | 0.01704 | 0.01014 | 42 |
| 14D | HEMOGLOBIN_max | 0.00160 | 0.00129 | 42 |
| 14D | HR_mean | 0.00520 | 0.00253 | 42 |
| 14D | HR_std | 0.00711 | 0.00603 | 42 |
| 14D | ISS_computed_max | 0.00102 | 0.00172 | 42 |
| 14D | ISS_notes_max | 0.02137 | 0.04692 | 42 |
| 14D | LACTATE_max | 0.00738 | 0.01124 | 42 |
| 14D | LEUKOCYTES_max | 0.00731 | 0.00590 | 42 |
| 14D | RESPIRATORYRATE_mean | 0.00246 | 0.00222 | 42 |
| 14D | RESPIRATORYRATE_std | 0.00160 | 0.00151 | 42 |
| 14D | SBP_mean | 0.00590 | 0.00464 | 42 |
| 14D | SBP_std | 0.00203 | 0.00135 | 42 |
| 14D | SPO2_mean | 0.00876 | 0.00790 | 42 |
| 14D | SPO2_std | 0.00401 | 0.00497 | 42 |
| 14D | TEG-LY30_max | 0.00024 | 0.00063 | 42 |
| 14D | TEG-MA_max | 0.00192 | 0.00513 | 42 |
| 14D | TEG-R_max | 0.00080 | 0.00208 | 42 |
| 14D | TEMP_mean | 0.00692 | 0.00388 | 42 |
| 14D | TEMP_std | 0.00293 | 0.00532 | 42 |
| 14D | static_cat:SEX | 0.09692 |  | 42 |
| 14D | static_cat:FIRST_HOSPITAL | 0.10743 |  | 42 |
| 14D | static_cat:A | 0.09887 |  | 42 |
| 14D | static_cat:B | 0.16384 |  | 42 |
| 14D | static_cat:C | 0.14968 |  | 42 |
| 14D | static_cat:D | 0.30107 |  | 42 |
| 14D | static_cat:HEIGHT_na | 0.02678 |  | 42 |
| 14D | static_cat:WEIGHT_na | 0.00627 |  | 42 |
| 14D | static_cont:AGE | 0.46460 |  | 42 |
| 14D | static_cont:ASMT_ELIX | 0.42819 |  | 42 |
| 14D | static_cont:HEIGHT | 0.25039 |  | 42 |
| 14D | static_cont:WEIGHT | 0.23168 |  | 42 |
| 14D | cat_ts:medication:anastethics | 0.02534 |  | 42 |
| 14D | cat_ts:medication:anti_thrombotic | 0.00879 |  | 42 |
| 14D | cat_ts:medication:antibiotics | 0.01114 |  | 42 |
| 14D | cat_ts:medication:blood | 0.00115 |  | 42 |
| 14D | cat_ts:medication:cardiovascular_drugs | 0.02911 |  | 42 |
| 14D | cat_ts:medication:diuretics | 0.02887 |  | 42 |
| 14D | cat_ts:medication:hemostatics | 0.03390 |  | 42 |
| 14D | cat_ts:medication:infusion | 0.01842 |  | 42 |
| 14D | cat_ts:medication:insulin | 0.00324 |  | 42 |
| 14D | cat_ts:medication:local_anastethics | 0.00628 |  | 42 |
| 14D | cat_ts:medication:neuro_drugs | 0.00997 |  | 42 |
| 14D | cat_ts:medication:opiods | 0.02629 |  | 42 |
| 14D | cat_ts:ADT:BED | 0.00747 |  | 42 |
| 14D | cat_ts:ADT:ICU | 0.00964 |  | 42 |
| 14D | cat_ts:ADT:OR | 0.00764 |  | 42 |
| 14D | cat_ts:ADT:TC | 0.01349 |  | 42 |
| 30D | BASE_EXCESS_max | 0.00359 | 0.00372 | 12 |
| 30D | EWS_SCORE_max | 0.00321 | 0.00346 | 12 |
| 30D | GCS_min | 0.00984 | 0.00510 | 12 |
| 30D | HEMOGLOBIN_max | 0.00084 | 0.00057 | 12 |
| 30D | HR_mean | 0.00414 | 0.00176 | 12 |
| 30D | HR_std | 0.00441 | 0.00188 | 12 |
| 30D | ISS_computed_max | 0.00125 | 0.00273 | 12 |
| 30D | ISS_notes_max | 0.02533 | 0.03689 | 12 |
| 30D | LACTATE_max | 0.00474 | 0.00734 | 12 |
| 30D | LEUKOCYTES_max | 0.00426 | 0.00235 | 12 |
| 30D | RESPIRATORYRATE_mean | 0.00494 | 0.00636 | 12 |
| 30D | RESPIRATORYRATE_std | 0.00235 | 0.00157 | 12 |
| 30D | SBP_mean | 0.00379 | 0.00262 | 12 |
| 30D | SBP_std | 0.00121 | 0.00052 | 12 |
| 30D | SPO2_mean | 0.00610 | 0.00669 | 12 |
| 30D | SPO2_std | 0.00279 | 0.00360 | 12 |
| 30D | TEG-LY30_max | 0.00161 | 0.00455 | 12 |
| 30D | TEG-MA_max | 0.00149 | 0.00280 | 12 |
| 30D | TEG-R_max | 0.00077 | 0.00095 | 12 |
| 30D | TEMP_mean | 0.00554 | 0.00283 | 12 |
| 30D | TEMP_std | 0.00940 | 0.01515 | 12 |
| 30D | static_cat:SEX | 0.09038 |  | 12 |
| 30D | static_cat:FIRST_HOSPITAL | 0.05445 |  | 12 |
| 30D | static_cat:A | 0.10820 |  | 12 |
| 30D | static_cat:B | 0.15396 |  | 12 |
| 30D | static_cat:C | 0.12133 |  | 12 |
| 30D | static_cat:D | 0.34608 |  | 12 |
| 30D | static_cat:HEIGHT_na | 0.00573 |  | 12 |
| 30D | static_cat:WEIGHT_na | 0.00160 |  | 12 |
| 30D | static_cont:AGE | 0.32453 |  | 12 |
| 30D | static_cont:ASMT_ELIX | 0.20949 |  | 12 |
| 30D | static_cont:HEIGHT | 0.27831 |  | 12 |
| 30D | static_cont:WEIGHT | 0.20460 |  | 12 |
| 30D | cat_ts:medication:anastethics | 0.01857 |  | 12 |
| 30D | cat_ts:medication:anti_thrombotic | 0.00495 |  | 12 |
| 30D | cat_ts:medication:antibiotics | 0.00705 |  | 12 |
| 30D | cat_ts:medication:blood | 0.00264 |  | 12 |
| 30D | cat_ts:medication:cardiovascular_drugs | 0.02299 |  | 12 |
| 30D | cat_ts:medication:diuretics | 0.03099 |  | 12 |
| 30D | cat_ts:medication:hemostatics | 0.03920 |  | 12 |
| 30D | cat_ts:medication:infusion | 0.01548 |  | 12 |
| 30D | cat_ts:medication:insulin | 0.00337 |  | 12 |
| 30D | cat_ts:medication:local_anastethics | 0.01304 |  | 12 |
| 30D | cat_ts:medication:neuro_drugs | 0.00742 |  | 12 |
| 30D | cat_ts:medication:opiods | 0.01450 |  | 12 |
| 30D | cat_ts:ADT:BED | 0.00781 |  | 12 |
| 30D | cat_ts:ADT:ICU | 0.00981 |  | 12 |
| 30D | cat_ts:ADT:OR | 0.01074 |  | 12 |
| 30D | cat_ts:ADT:TC | 0.01116 |  | 12 |

**Table S7.** Cohort-level mean absolute SHAP values across evaluation timeframes (1 hour, 6 hours, 12 hours, 1 day, 3 days, 7 days, 14 days, and 30 days) for all input features, computed on active-cohort holdout patients using GradientExplainer with a background dataset of 1000 training patients. Features are grouped by input modality: continuous time series, static categorical, static continuous, and categorical time series. For continuous time series features, standard deviations of absolute SHAP values across patients are reported. Sample sizes at each timeframe reflect the number of active-cohort patients evaluated (n reported per timeframe).

# References

1.	CUDA Toolkit Archive. NVIDIA Developer. Available from: https://developer.nvidia.com/cuda-toolkit-archive [accessed Jan 23, 2025]

2.	Paszke A, Gross S, Massa F, Lerer A, Bradbury J, Chanan G, Killeen T, Lin Z, Gimelshein N, Antiga L, Desmaison A, Kopf A, Yang E, DeVito Z, Raison M, Tejani A, Chilamkurthy S, Steiner B, Fang L, Bai J, Chintala S. PyTorch: An Imperative Style, High-Performance Deep Learning Library. Advances in Neural Information Processing Systems Curran Associates, Inc.; 2019. Available from: https://papers.nips.cc/paper_files/paper/2019/hash/bdbca288fee7f92f2bfa9f7012727740-Abstract.html [accessed Jan 23, 2025]

3.	Pedregosa F, Varoquaux G, Gramfort A, Michel V, Thirion B, Grisel O, Blondel M, Prettenhofer P, Weiss R, Dubourg V, Vanderplas J, Passos A, Cournapeau D. Scikit-learn: Machine Learning in Python. MACHINE LEARNING IN PYTHON.

4.	Lundberg SM, Lee S-I. A Unified Approach to Interpreting Model Predictions.

5.	Akiba T, Sano S, Yanase T, Ohta T, Koyama M. Optuna: A Next-generation Hyperparameter Optimization Framework. Proceedings of the 25th ACM SIGKDD International Conference on Knowledge Discovery & Data Mining New York, NY, USA: Association for Computing Machinery; 2019. p. 2623–2631. doi: 10.1145/3292500.3330701

6.	Traumemanual og Akutkaldsmanual. Available from: https://www.rigshospitalet.dk/afdelinger-og-klinikker/hovedorto/traumecenter-og-akut-modtagelse/for-fagfolk/Sider/primaere-visitationskriterier.aspx [accessed Mar 3, 2025]
